# TBISTAT: An open-source, wireless portable, electrochemical impedance spectroscopy capable potentiostat for the point-of-care detection of S100B in plasma samples

**DOI:** 10.1371/journal.pone.0263738

**Published:** 2022-02-07

**Authors:** Francisco Burgos-Flórez, Alexander Rodríguez, Eliana Cervera, Valtencir Zucolotto, Marco Sanjuán, Pedro J. Villalba

**Affiliations:** 1 Department of Medicine, Biotechnology Research Group, Universidad del Norte, Barranquilla, Colombia; 2 Department of Mechanical Engineering, Rational Use of Energy and Preservation of the Environment Group (UREMA), Universidad del Norte, Barranquilla, Colombia; 3 GNano–Nanomedicine and Nanotoxicology Group, São Carlos Institute of Physics, University of São Paulo, São Carlos, São Paulo, Brazil; Pandit Deendayal Petroleum University, India, INDIA

## Abstract

Point-of-Care (POC) testing for biomarker detection demands techniques that are easy to use, readily available, low-cost, and with rapid response times. This paper describes the development of a fully open-source, modular, wireless, battery-powered, smartphone-controlled, low-cost potentiostat capable of conducting electrochemical impedance spectroscopy for the electrochemical detection of the S100B protein captured in an ANTI-S100B functionalized thin-film gold interdigitated electrode platform to support traumatic brain injury diagnosis and treatment. EIS results from the developed potentiostat were validated with a commercial benchtop potentiostat by comparing impedance magnitude and phase values along the EIS frequency range. In addition, an experimental design was performed for detecting S100B in spiked human plasma samples with S100B concentrations of clinical utility, and a calibration curve was found for quantifying S100B detection. No statistically significant differences were found between EIS results from the developed potentiostat and the commercial potentiostat. Statistically significant differences in the changes in charge transfer resistance signal between each tested S100B concentration (p < 0.05) were found, with a limit of detection of 35.73 pg/mL. The modularity of the proposed potentiostat allows easier component changes according to the application demands in power, frequency excitation ranges, wireless communication protocol, signal amplification and transduction, precision, and sampling frequency of ADC, among others, when compared to state-of-the-art open-source EIS potentiostats. In addition, the use of minimal, easy acquirable open-source hardware and software, high-level filtering, accurate ADC, Fast Fourier Transform with low spectral leakage, wireless communication, and the simple user interface provides a framework for facilitating EIS analysis and developing new affordable instrumentation for POC biosensors integrated systems.

## Introduction

Traumatic brain injury (TBI) is a brain dysfunction produced by an external force, usually due to a sudden movement or blow to the head. Delays in the clinical identification of neurological impairment during the acute phase of TBI lead to higher mortality among patients [1]. This delay could often be related to the subjective and qualitative nature of the Glasgow Coma Scale (GCS), a technique routinely used as a strategy to classify the TBI severity [2]. In addition to the GCS, other invasive and non-invasive neuromonitoring techniques help to establish criteria for medical decisions. However, they require expertise and advanced medical skills and demand high costs for healthcare systems, limiting their availability in many resource-constrained environments, as in low-middle income countries, where the clinical examination is, in many cases, the only accessible tool for neuromonitoring [3].

S100B, a calcium-binding dimeric protein (MW: 21 KDa) expressed in astrocytes and found in very low levels under physiological conditions in serum/plasma, has been previously suggested as a potential biomarker for TBI [4, 5]. Following a TBI, S100B is released from damaged nerve cells into the bloodstream by passing through the blood-brain barrier (BBB), which could be disrupted after head primary injury [6]. The clinical significance of S100B depends on the type and severity of the brain damage [7]. Various cutoff values of S100B have been proposed for identifying brain injury [8]. A cutoff level of 100pg/mL [9] has been used in the mild TBI to discard the presence of intracranial hemorrhages in the CT scans, and values closer to 30 pg/mL have been reported as an indicator of BBB permeability even with no associated symptoms [10]. Likewise, patients with moderate to severe TBI could display higher serum/plasmatic S100B levels in the order of ng/mL, which correlates to intracranial hypertension, neurological worsening, and poor response to treatment [7]. In the clinical context, the measurement of biomarkers such as S100B demands a technique that is easy to use, readily available, low-cost, and with a rapid response time, ideally at the Point-of-Care (POC).

The advances in material science, nano-fabrication, molecular biology, and immunology have supported the continuous generation of new POC electrochemical biosensors capable of detecting a myriad of biological substances of interest in medicine [11–15]. Among electrochemical detection techniques, electrochemical impedance spectroscopy (EIS) has been one of the most widely used for impedance measurements due to its minor system interference, fast response, and accurate, reliable results [16]. EIS has been effectively used for affinity biosensors [17–21] since it can monitor events such as antigen-antibody binding (Ag-Ab) that occur on the surface of the electrodes, where small changes in impedance are proportional to the concentration of the measured antigen.

To fulfill the objectives of POC technology, the development of portable, precise, minimal sample preparation requirements, high-quality and cost-effective instrumentation, and techniques that allow the detection of the analytes such as S100B are essential [22]. Researchers in need of performing precise analytical EIS tests for biosensing applications often employ a benchtop potentiostat, a highly-priced (more than USD 10,000), heavy, patented, commercial device. Current electronics technology has enabled simplifying the large, heavy, and expensive benchtop potentiostats. Several companies, such as Gamry, PalmSens, Ivium, and Metrohm, have developed portable variants of their benchtops with similar specifications but with prices still above USD 1,000. As explained by Dryden et al. [23], commercial potentiostats function as black boxes, as they give limited information about their hardware and software technical specifications, which can make the development of new measurement techniques and integration with other instruments challenging for researchers, educators, and for further product integration [24]. In addition, current potentiostat prices still prohibit their widespread use, specifically in resource-limited settings. Still, most efforts in the literature have been devoted to biosensor miniaturization, increased sensibility, and reproducibility, whereas commercial potentiostats have remained expensive, requiring time-consuming tasks for adequate configuration [25].

Various research groups have embraced the task of developing low-cost EIS-capable portable potentiostats which can be available to the general public for their replication and adaptation to their own needs (Table 1). To our knowledge, potentiostats made by Jenkins et al. [22], Jiang et al. [12], Pruna et al. [26], and Zhang et al. [27] are the only ones whose functionality allows EIS. Even though considerable details have been given for the above prototypes, only Jenkin’s et al. [22] ABE-Stat provides wireless smartphone-controlled electrochemical detection and fully documented open-source hardware and software. A portable potentiostat with wireless connectivity to smartphones would facilitate electrochemical analysis at the POC and in emergencies at remote locations, where access to a computer or wired connection to a device is unlikely, such as the case of S100B detection soon after TBI occurs. In addition, technology developed in an open-source format allows researchers to quickly adopt the device’s design and develop specific-purpose potentiostats without the need to design and test the electronics from scratch [24].

**Table 1:** Feature comparison of literature reviewed potentiostats with the proposed device.

Reference	EIS	2–3 Electrode Systems	Android integration	Independent Impedance circuit	Wireless	Windows/ Linux Integration	Fully documented open-source hardware and software
**Punter-Villagrasa (2014)** [28]	✓	✓	×	×	×	✓	×
**Zhang (2016)** [27]	✓	✓	✓	×	✓	×	×
**Pruna (2017)** [26]	✓	×	×	×	×	✓	×
**Ainla (2018) UWED** [24]	×	✓	×	×	✓	×	×
**Jiang (2019)** [12]	✓	✓	×	✓	×	✓	×
**Jenkins (2019) ABE-Stat** [22]	✓	✓	✓	×	✓	×	✓
**Proposed device**	✓	✓	✓	✓	✓	×	✓

✓Feature is present on the potentiostat. × Feature is not present on the potentiostat.

It should be noted that the only current truly open-source potentiostat capable of performing EIS is the one developed by Jenkins et al. [22]. However, as the authors indicate, there are still drawbacks in the precision and generation of reliable results during electrochemical impedance measurements. First, the presence of discontinuities at characteristic frequencies (2 Hz, 60 Hz, and 2 kHz) is likely due to the way ABE-stat calculates impedance and instabilities associated with the control amplifier during small-signal measurements. Authors also describe the need for a more robust microcontroller unit (MCU) that provides a higher number of general-purpose input-output ports (GPIOs) and a less noise-prone Bluetooth module (BM), ideally a Bluetooth low energy one (BLE). In addition, the use of the AD5933 as impedance analyzer with a 1024-points single-frequency 16 MHz clock-dependent Discrete Fourier Transform (DFT) causes higher noise and lower precision in EIS analyses, given its nonlinear nature, and prevents accurate impedance determination at frequencies below 1 kHz. Furthermore, higher errors due to spectral leakage are found if evaluated frequencies do not correspond to integer numbers of cycles over the sampled period. The authors also express the inability of the device to generate coherent results for nonlinear non-resistive loads, which is critical when making Nyquist plots of electrochemical systems, considering their capacitive nature. Therefore, the definition of circular regions of the said diagram is significantly affected, as greater inaccuracies in charge transfer resistance (RCT) values might show up. RCT depends on the change in the capacitance of the bio-functionalized surface of the working electrode, mainly given by the number of antigen-antibody bonds formed after the sample is applied. Therefore, increasing the device’s precision for its use in affinity biosensors such as the one employed in this work is necessary.

For the above reasons, this paper describes the development of a fully open-source modular, low-cost, portable potentiostat capable of performing EIS for the electrochemical detection and quantification of the S100B protein biomarker captured in a two-electrode platform, referred to from now on as TBISTAT. The overall design approach is inspired by the component selection, layout, and firmware design of Ainla [24] and Jenkins [22]. However, the design has been made modular to allow easy component changes according to the application demands in power, frequency excitation ranges, wireless communication protocol, signal amplification and transduction, precision, and sampling frequency of analog digital conversion (ADC), among others. TBISTAT features an independent impedance analyzer circuit, using the AD5933 as only a signal generator with a programmable external clock, and can be interfaced with both classic Bluetooth and BLE modules. In addition, the true 12-bit inbuilt ADC of its MCU and active hardware and digital filter reduces current variance during ADC. TBISTAT is also controlled by an Android smartphone application that performs a 1000-point real Fast Fourier Transform (FFT) of a non-integer number of cycles over sample periods to achieve low-spectral leakage transformation for logarithmic frequency points.

The TBISTAT functionality is assessed and validated by conducting EIS on three experimental systems: An AUTOLAB dummy cell circuit composed of a 100 Ω resistor in series with the parallel circuit of a 1μF ceramic capacitor and a 1 KΩ resistor; Bare thin-film gold interdigitated electrodes (AUIDEs) drop casted with 10uL of 10mM K3[Fe(CN)6] in 0.2M KCl solution; ANTI-S100B functionalized thin-film AUIDEs exposed to 316 pg/ml of S100B spiked human plasma samples, and drop casted with 10uL of 10mM K3[Fe(CN)6] in 0.2M KCl as support solution. EIS results from the developed potentiostat were validated with an Autolab/M204 benchtop potentiostat by comparing impedance magnitude and phase values along the EIS frequency range and through a T-test that compared means of changes in capacitance values (ΔC) using single frequency analysis (SFA). In addition, an experimental design was performed for detecting S100B in spiked human plasma samples with S100B concentrations of clinical utility (31pg/mL, 100 pg/mL, and 316pg/mL) using the change in charge transfer resistance (ΔRCT) as the response variable. A one-way Welch ANOVA was applied to check for differences between treatment groups (S100B concentrations), followed by Games-Howell as a post-hoc test. A regression model (calibration curve) was developed together with a lack of fit test to determine model adequacy to the response variable.

The use of minimal, modular, easy acquirable open-source hardware and software, together with high-level filtering, low-cost, accurate ADC, smartphone-executed FFT, adequate analog/digital ground/power planes, wireless communication, and a simple user interface, makes the TBISTAT a framework for facilitating EIS analysis for POC applications such as S100B detection and provides new opportunities for the development of affordable diagnostics, sensors, and wearable devices. To our knowledge, this is the first portable potentiostat entirely built for measuring S100B concentration on plasma samples using EIS. The open-source nature of this work encourages users to optimize this design for their purposes and needs.

## Materials and methods

### Ethics statement

The study was conducted according to the guidelines of the Declaration of Helsinki, and approved by the Ethics Committee of UNIVERSIDAD DEL NORTE, Barranquilla-Colombia (act number 167, approved on 25-01-2018).

### Electrodes and reagents

AUIDEs with 180 pairs of interdigitated gold electrodes (5/5 μm, electrode/gap) were obtained from Micrux Technologies, Spain. Bovine Serum albumin (BSA), 1-ethyl-3-(3-dimethylaminopropyl) carbodiimide (EDC), phosphate-buffered saline (PBS), 30% Hydrogen Peroxide, 30% Ammonium Hydroxide, and potassium ferrocyanide (III) powder <10um, 99% (702587) were obtained from Sigma-Aldrich (US). Cysteamine >98.0% (30070) was purchased from Sigma-Aldrich (Brazil). Potassium chloride (P217500) ACS 99.0 a 100% was obtained from Fisher (US). All reagents used were of analytical grade. Deionized water (DW) (MiliQ®) was employed to prepare all solutions. Recombinant Anti-S100B antibody [EP1576Y]—Astrocyte Marker and Recombinant Human S100B protein (ab55570) were acquired from Abcam (US).

### Biosensor construction

**Fig 1:**
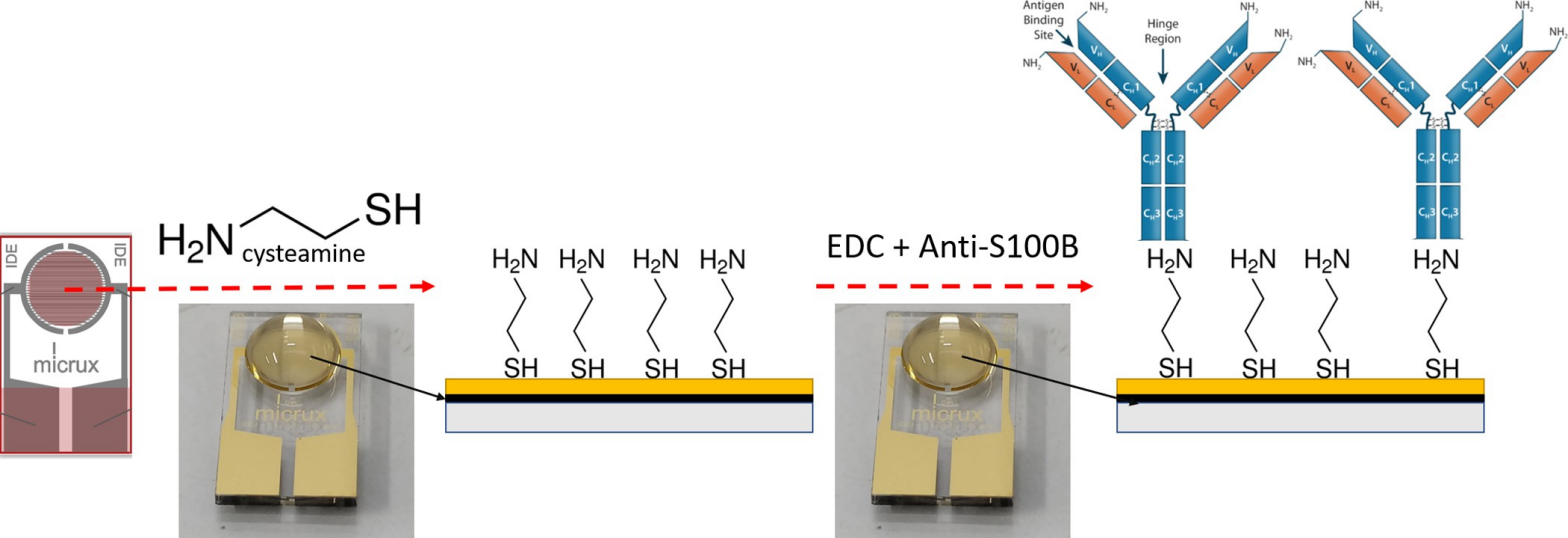
Graphical representation of the covalent immobilization of anti-S100B onto AUIDEs using a cysteamine/EDC approach.

AUIDEs biosensor construction comprised three consecutive processes: cleaning, self-assembled monolayer (SAM) formation, and surface functionalization (Fig 1). AUIDEs were first thoroughly cleaned following RCA-1 cleaning protocol by immersion in a 5:1:1 DW, 27% NH4OH, 30% H2O2 solution for one minute, and then generously rinsed with DW and dried using N₂. An EIS essay was performed in potassium ferrocyanide to assess the cleaning efficiency of AUIDEs working surface, which were again rinsed with DW and dried using N₂. For the SAM formation, ten microliters of 0.5M cysteamine in PBS were drop-casted on the AUIDEs WE surface and left covered at room temperature (RT) for one hour on a rotary machine to improve Au-cysteamine molecular interactions. AUIDEs were then rinsed with DW and dried using N₂. Subsequently, a solution of 0.5 M EDC in 10mM PBS (pH 7.4) with anti-S100B at 50ug/ml was prepared and vortex mixed every 15 minutes during two hours at RT for anti-S100B carboxyl group activation. The zero-length cross-linking functionalization of anti-S100B on cysteamine-modified AUIDEs was performed by drop-casting ten microliters of the EDC-anti-S100B solution on the AUIDE WEs surface, which were then left covered at RT for 12 hours on a rotary machine for anti-S100B conjugation to the Au-cysteamine SAM. Electrodes were then carefully rinsed with DW and dried using N₂. An EIS scan was then performed with potassium ferrocyanide to characterize the electrochemical behavior of the functionalized WEs. AUIDEs WEs were then blocked with 10 microliters of 0.5% BSA in 10 mM PBS and left covered at RT overnight on a rotary machine. Electrodes were then thoroughly rinsed with DW and dried using N₂ at RT, and an EIS was done in potassium ferrocyanide to set a single reference RCT baseline for each AUIDE. Small reagent concentrations were used in this work to reduce manufacturing costs. In addition, to avoid undesirable fixation of reagents to the recipient walls, low retention tips and tubes were used to dilute, aliquot, and drop-casting antibodies and crosslinker molecules during functionalization and further S100B tests.

### Preparation of spiked human plasma samples

Human whole blood was obtained from a venipuncture of a healthy donor with previous written consent under the Universidad del Norte (Barranquilla, Colombia) ethics committee No. 167. Whole blood, collected to Gel & EDTA K2 tubes (Improvacuter® 722350202), was then centrifuged for 30min at 10,000g to remove proteins with molecular weight (MW) over 30 KDa. Human plasma was extracted by pipetting and aliquoting in low retention tubes. Each aliquot was spiked with corresponding amounts of the S100B protein to obtain aliquots of 31, 100 and 316 pg/mL and, after resuspended, stored at -20°C in low retention tubes. Each spiked human plasma sample was thawed 15 min before the test and resuspended to be drop-casted on the AUIDE WE surface.

### High-level representation of potentiostat system

**Fig 2:**
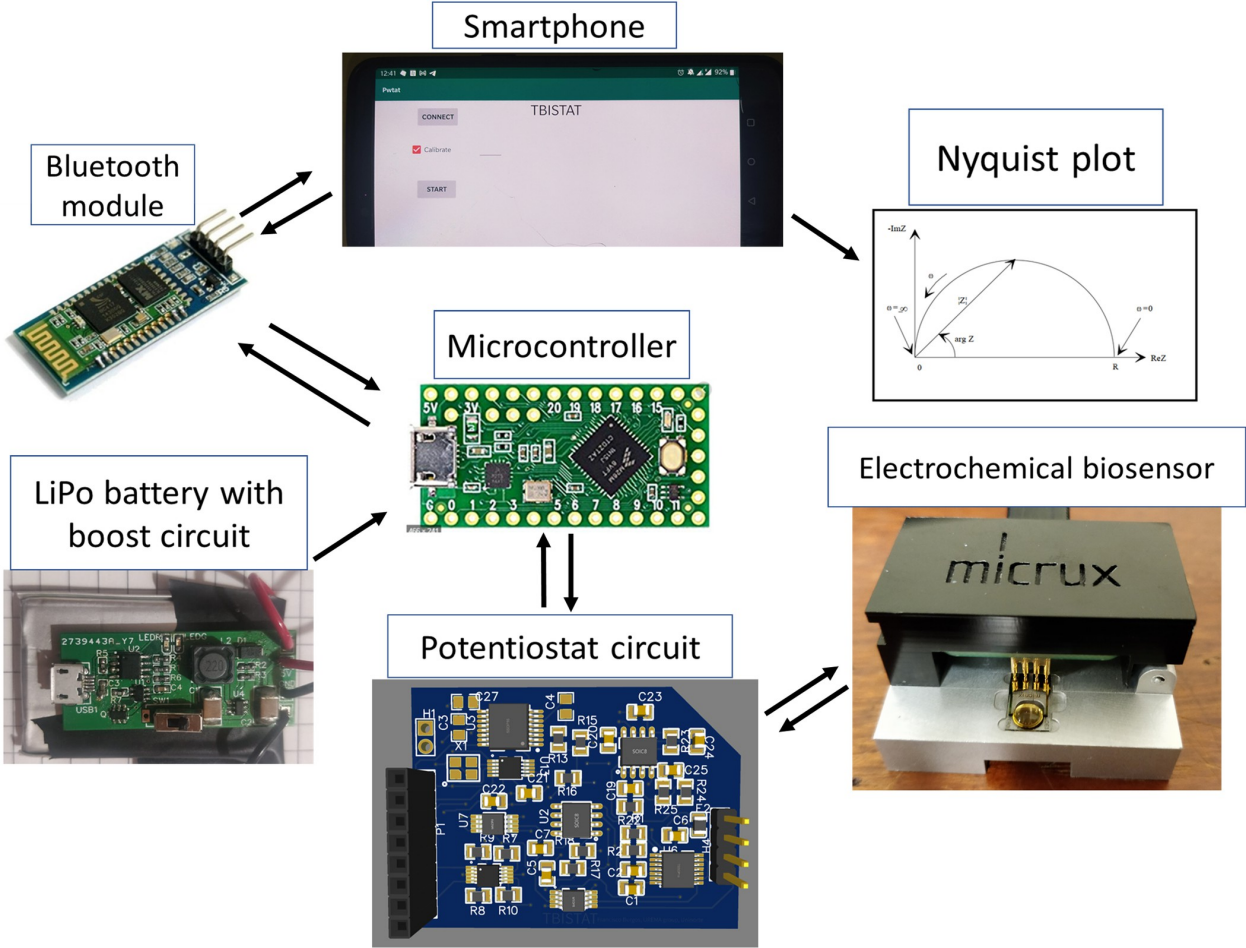
General representation of the system components.

Fig 2 shows a general representation of the system. The proposed design features a lithium-ion polymer battery (LiPo) with a boost circuit that powers a Teensy LC MCU and an HC06 Bluetooth 2.0+EDR (B2.0) module. The MCU powers a 3.3 V digital-analog front-end potentiostat circuit (PC), which interfaces with an Android application running on a smartphone (or a tablet) using the BM connected to the MCU. The smartphone provides a user interface to wirelessly connect with the MCU via Bluetooth, calibrate the device impedance measurements, perform EIS on the electrochemical two-electrode biosensor platform, and display the impedance measurements in a real-time Nyquist plot as EIS experimental runs take place on the biosensor platform. During an EIS measurement, the PC excites the electrochemical cell, which in turn generates an electrochemical output current (EOC) which is transduced and amplified using a transimpedance amplifier (TIA) and acquired with the Teensy LC ADC. The Teensy LC MCU sends digitalized acquisitions to the smartphone app via Bluetooth, and a real-time FFT of the incoming data is performed in the Android application, which results in amplitude and phase data for each frequency point in the EIS frequency spectrum. The Nyquist plot is constructed in real-time as each FFT is done during the excitation of the electrochemical biosensor system. The S100B concentration is obtained by calculating the ΔRCT from the EIS using a regression model which relates ΔRCT to the S100B concentration. Once EIS is completed, the Android application shows the value of S100B concentration and information regarding possible brain injury related to the found S100B concentration.

### Hardware design

#### LiPo battery boost, charger, protection circuit module

A 3.7 V, 1000 mAh lithium-ion polymer battery (LiPo) was used as a power supply for the BM and MCU. The PC is powered by a 3.3 V low-dropout regulator (LDO) inherent to the MCU. Considering that the MCU and BM require a 5 V voltage supply and that LiPo batteries are rechargeable and have a significant risk of igniting if a short circuit happens, a boost, USB charger, short-circuit protection circuit was employed to fulfill these purposes. Additional details are found in S1 File.

#### Wireless communication with Bluetooth module

The TBISTAT communicates with a smartphone or a tablet using either the wireless BLE protocol or B2.0. A host program in the smartphone receives the input parameters of the experiment from the user, communicates the experimental protocol to the TBISTAT, receives the raw data (ADC) of the EIS measurement, and visualizes it for the user in a real-time Nyquist plot. The modules HC06 and HM-10 were used for performing wireless communication between TBISTAT and a smartphone.

Communication between BMs and MCU is done using the universal asynchronous receiver/transmitter (UART) interface, which is a block of circuitry responsible for implementing serial communication using two channels, one for transmission (Tx) and one for reception (Rx). Both modules permit serial transfer as high as 115200 Bauds. Additional details about the BMs technical specifications are found in S1 File.

#### MCU

A Teensy LC MCU was employed as the control unit for the developed potentiostat. This MCU features an ARM Cortex-M0+ processor at 48 MHz, 62K Flash, 8K RAM, 12-bit analog input & output, hardware Serial, 400 KHz I2C, USB, and a total of 27 Input-output (IO) pins. Teensy LC flash memory programming is achieved by the Teensy Loader application, which is compatible with the popular open-source Arduino IDE. Hence, code can be written and compiled in Arduino and automatically uploaded using the Teensy loader application, requiring only a USB connection between the MCU and a computer.

Teensy LC processor speed and 12-bit ADC allows interrupt-driven ADC with sampling rates as high as 200 KHz, greatly surpassing the sampling criterion defined in the Nyquist–Shannon sampling theorem for a 10 KHz signal [29]. In addition, the presence of multiple Digital IOs and fast UART allows controlling multiple analog switches and fast serial transfer. The 8K RAM allows allocation of up to 2000 unsigned 16-bits integers for each EIS frequency measurement point for sufficient FFT frequency resolution [30]. I2C communication is employed for controlling the SI5351 clock generator IC and the direct digital synthesizer (DDS) AD5933 IC. The former capabilities are not fully met by Ainla et al. RFDUINO MCU [24] or Jenkins et al. ESP12S MCU [22] without including additional ICs (like peripheral ADC IC or addition I/O ports for ESP12S, or MCU change for higher processor speeds (RFDUINO)) which increase manufacturing costs and device size. The circuit schematics and design description of the MCU module board are found in S1 File.

#### Digital-analog front-end potentiostat circuit

**Fig 3:**
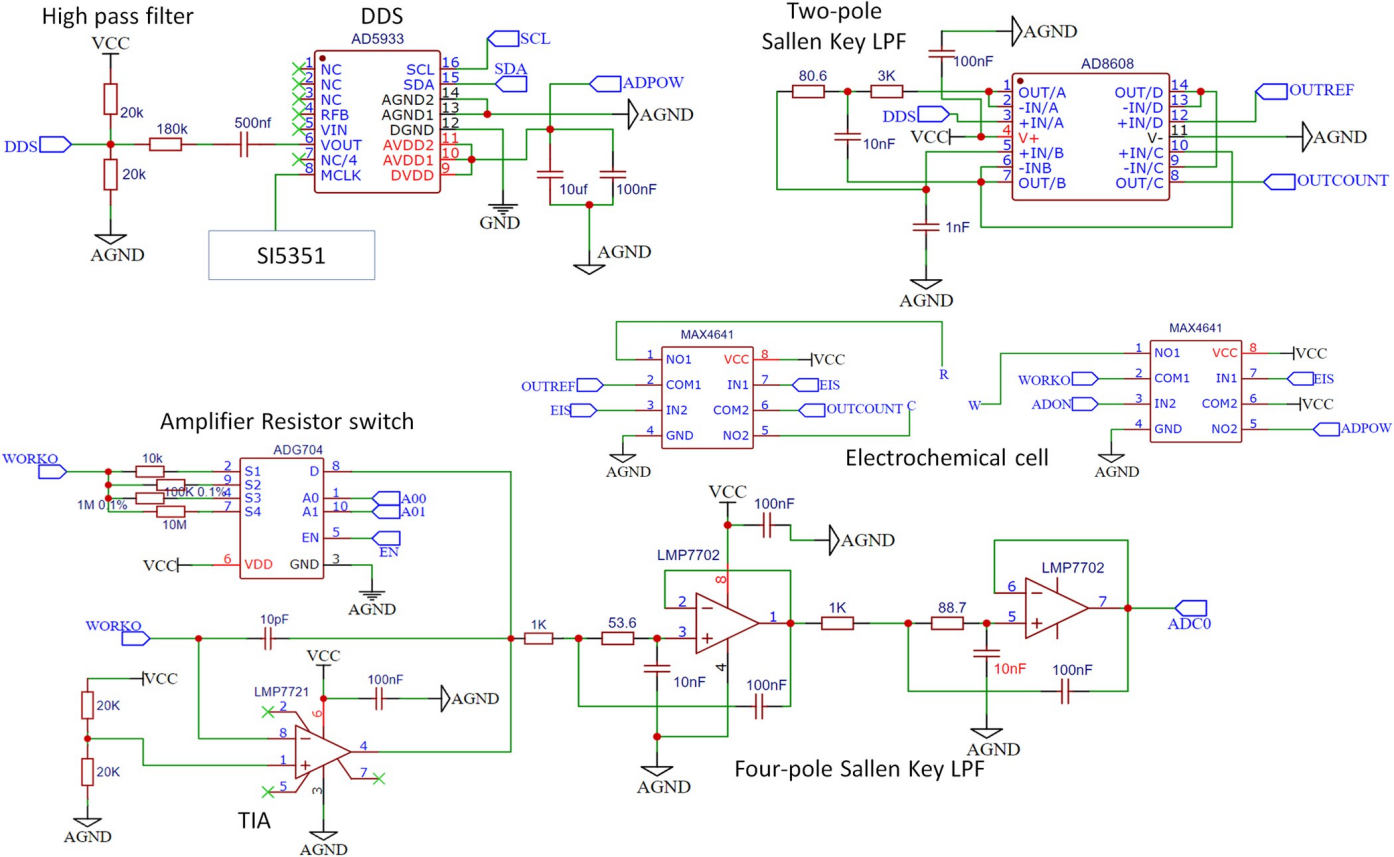
Potentiostat circuit with TIA circuit. Analog switches (MAX4641 and ADG704) are controlled by Teensy LC (not shown) and isolate the electrochemical cell. The TIA gain is controlled by one of four resistors selected by the ADG704 switch.

Fig 3 shows an overall circuit schematic of the PC. The digital-analog front-end circuit comprises three modules: The AC excitation signal design module made up of the clock generator IC, the DDS, and the signal conditioning circuit; The PC module made up of analog switch ICs, TIA circuit, and signal filtering circuit; and the mixed-signal grounding module.

The generation of an AC signal from DC voltage sources relies on the adequate use of a DDS IC. Similar to previous studies [22, 27], The TBISTAT employs the AD5933 network analyzer IC, which consists of a 27-bit direct digital synthesis (DDS) sine excitation voltage generator, a digital-to-analog converter (DAC), and a programmable gain amplifier (PGA) which determines the AC signal amplitude in four possible ranges [31].

The AD5933 can produce 1KHz-100KHz AC signals without any external components. For the excitation signal frequency to go below 1 kHz, the clock that drives the AD5933 must be scaled down. Similar to the implementation made by Zhang et al. [27], this work uses the S5351A IC as a clock generator connected to the external clock pin on the AD5933, which allows an excitation bandwidth increase between 1 Hz - 100Khz.

During EIS, electrochemical systems are commonly perturbed with AC excitation voltages of about 5 to 10 mVp to preserve the linear behavior of the EOC [32]. Potentials as small as 2 mVp and as large as 20 mVp have been employed [33]. The AD5933 can produce AC signals with four preprogrammed AC amplitudes and DC-bias voltages which, if not corrected, could induce polarization across the electrochemical cell under test and significantly alter the impedance response of the system by inducing a DC component in the EOC [31]. Therefore, a high pass filter/amplitude reduction circuit was designed to cancel the DC component and achieve a 10 mVp, 3.3/2 VDC, biased excitation signal. This circuit induces a DC bias of 3.3/2 V to provide a reference DC voltage via a simple voltage divider circuit, also known as virtual ground voltage (VGV). This feature allows the AC signal component to swing around the middle of the 0–3.3V range for a unipolar power supply (0 to 3.3V). An operational amplifier (OAMP) was used as a buffer of the filtered signal and for low output impedance (less than 1 Ω). In this way, using the above circuit, only one OAMP is needed to attain filtering, amplitude reduction, VGV, and low output impedance of the excitation signal. To isolate high-frequency harmonics from wireless communication and the AD5933 MCLK itself, a 100 KHz two-pole SKLPF was implemented in a second OAMP circuit.

Two high-speed, low-voltage analog switches (MAX4641 IC) are employed and controlled by the EIS digital control signal coming from the MCU for isolating the electrochemical cell counter, reference, and working electrodes. During an EIS measurement, EIS output is set high, which closes the MAX4641 analog switches, and the AC excitation signal perturbs the electrochemical cell. Even though the AD5933 IC is capable of performing transduction of the EOC through an embedded TIA and executing a DFT itself for the obtention of phase and magnitude values of the analyzed impedance, previous works have noted the presence of significant errors arising from DC and AC spectral leakage given by the limitations of the AD5933 impedance analyzer implementation [31, 34–37]. Among them, discontinuities in the test phasor, DC and AC leakage, and overall inability to measure impedance at low-frequency signals (less than 2 KHz) for nonlinear non-resistive loads have been reported, which are crucial since they usually correspond to circular portions of Nyquist plots in many electrochemical systems. AD5933 performs a single-point DFT, meaning that the analysis or correlation frequency in its core is always at the same frequency as the current output excitation frequency. If the input signal period over the 1024-point sample interval is an integer, there will be a smooth transition from the end of one period to the beginning of the next one. However, if this is not met, there will not be a smooth transition between beginning and end, and spectral leakage will ensue. As explained by Matsiev et al. [37], the errors reported in the literature are erroneously attributed to spectral leakage and could, instead, be the result of the discontinuity in the test phasor, which induces DC and AC leakage. The conventional calibration produces a single multiplicative gain factor that leads to substantial errors and undue disappointment in the device performance [36], especially at the lower end of the operating frequency range, which could explain the unreliability of Jenkins et al. ABE-Stat at frequencies below 2 KHz [22].

For the above reasons, this work discards using the AD5933 impedance analyzer circuit and proposes that the EOC is transduced by an electrometer grade amplifier (LMP7721 IC), whose output voltage amplitude is controlled by one of four resistors selected by the analog multiplexer ADG704 IC. Similar to the high-pass filter circuit used in the AC excitation signal design module, a 3.3/2 V VGV is fixed on the positive input of the TIA via a simple voltage divider circuit. This circuit ensures low DC bias between the input excitation signal and output voltage of the TIA, which is a sufficient condition for EIS of the ANTI-S100B functionalized AUIDEs to detect S100B, as seen in Rodriguez et al. [38]. The TIA voltage signal passes through a fourth-order 10 KHz SKLPF for high-frequency noise filtering using an LMP7702 OAMP IC. The cutoff frequency is determined following the EIS measurement specifications established by Rodríguez et al. [38] for S100B detection, and the ADC conversion is performed using the MCU ADC. Additional details about the PC design are found in S1 File.

### Software design

An Android application was developed to interface with the MCU firmware over either B2.0 or BLE protocols. The used libraries have GNU General Public License v3, which permits unrestricted use for software development.

Using the firmware developed for RFDUINO by Ainla et al. [24] as a reference, the Teensy LC main loop is equipped to listen, interpret, and execute the commands sent by the smartphone in the string format. The MCU serial module receives serial commands in the above format from the Android application for setting AD5933 external clock frequency, number of FFT points to acquire, TIA gain resistance, DDS word for AC excitation, sampling frequency, and initializing EIS scans.

The user interface of the Android application comprises only two buttons (connect and start), a checkbox for calibration, and a text input space for selecting calibration resistance value. The user initially presses the connect button to establish a Bluetooth connection between the smartphone and the BM connected to the MCU. When the start button is pressed, the smartphone configures MCU for EIS and starts measuring each frequency point. If the Calibration checkbox is checked before pressing start, the user must input a number corresponding to the calibration resistance to be used. Then, the smartphone performs calibration of system phase and magnitude for each EIS frequency for the defined calibration resistance. When data coming from BM is received, an FFT is performed. If the user is performing calibration, the magnitude and phase are stored on an excel file. If the user is performing a conventional measurement to detect the S100B biomarker, a Nyquist plot is displayed in real-time as FFT on each EOC received data is performed. Once EIS is finished, ΔRCT of the EIS is calculated for finding S100B concentration, which is shown together with TBI diagnosis support information. Additional details of the software implementation of the EIS measurements are discussed in S2 File, while source code for MCU firmware and Android Studio application files can be found in S3 File.

### S100B regression model and TBI support information

There is no easy way to obtain RCT from a Nyquist plot since it is difficult to define an electrochemical model that accurately fits the impedance data. Only proprietary software for this purpose is found in the literature, which would restrict TBISTAT use. Hence, a simple linear regression model of the lowest three frequency points of the Nyquist plot is performed to obtain an approximate RCT at y = 0. The calibration model of ΔRCT vs S100B using AUIDEs is used for determining sample S100B concentration. The value of the S100B concentration is then shown together with TBI classification and treatment support information for the health care professionals, which is based on previously reported results about the clinical utility of S100B for diagnosis and treatment of TBI [4, 5].

### Prototype fabrication

Circuit schematics and printed circuit board (PCB) designs were made in the free web-based electronic design automation (EDA) suite EasyEDA. The PCB designs can be found in S1 File. PCB fabrication was done by JLCPCB (China) upon sending the Gerber files of each designed PCB. Gerber files and schematics can be found in S4 File.

A surface mount technology (SMT) screen printing stencil was also ordered from JLCPCB for each PCB. Circuit components were acquired from Digikey (USA), LCSC (China), and AliExpress (China). PCB passive components assembly was performed via manual soldering, while ICs were soldered by screen printing liquid solder using the provided SMT stencils, followed by heating with a temperature-controlled hot air gun of a VIVO HOME 892D solder rework station (USA). The bill of materials (BOM) for each PCB is found in S4 File.

### System calibration and impedance measurement

Calibration of each TBISTAT is done on the assumption that system phase and magnitude are properties that depend on the accuracy of the VGV used for EIS (≈ 1.65 V for 3.3 V DC supply), the frequency response of the PC for the excitation signal frequency range, and the intrinsic variability of the functionality of the passive and active components that constitute the PC. In order to find system impedance, one must measure the magnitude and phase of the EOC produced by a two-terminal cell with a known resistor value since it does not alter the system phase as it is a non-capacitive or inductive load. Using the FFT, TBISTAT estimates a signal magnitude and phase of the frequency bin corresponding to each AC excitation signal frequency. This magnitude is stored and related to the resistor value and the measured phase. Care must be taken to ensure that the signal magnitude acquired for the defined resistor is unsaturated (less than 1.6 AC voltage amplitude with respect to reference VGV) and shows a high signal-to-noise ratio (at least 50 mV of AC voltage amplitude with respect to VGV). Calibration must be done using as many resistor values as possible to achieve high precision throughout the impedance detection range for all available settings [22]. In this work, 103.5 Ω,141.9 Ω, 238 Ω, 393 Ω, 601 Ω, 1183 Ω, 1360 Ω, 1830 Ω, 2960 Ω, 5530 Ω, 11330 Ω, 12980 Ω, 15600 Ω, 18200 Ω, 24300 Ω, and 33200 Ω resistors were used for calibration. Resistors of 1183 Ω and 11330 Ω are used for both the upper and lower limits of impedance measurement ranges of the 10 KΩ and 100 KΩ TIA resistances, respectively. Hence, a total of 18 resistors were employed for calibration. Therefore, calibration of the impedance measurements (system magnitude and phase) was done for a total of 18 different settings (one possible AC amplitude peak of 10 mVp, three different TIA gain resistances for measuring impedances between 100 Ω and 35 ΩK, and six different resistors for each TIA gain resistance). A calibration file example for the mentioned resistor values is found in S5 File.

Once calibration has been performed, the user can press start on the Android Application, which will trigger EIS measurements and real-time Nyquist plot construction for the electrochemical cell under test. Additional details about TBISTAT calibration and EIS measurements can be found in S1 File.

### Electrical characterization

#### Power requirements

Power consumption was estimated by measuring current draw from a fully charged (3.7 V) 1000 mAh LiPo battery using a Fluke 117 handheld multimeter (USA) for both standby and active EIS measurements of a 100 Ω resistor connected between the working and counter/reference electrodes in a two-electrode configuration.

#### Electrical noise

TBISTAT noise was evaluated by performing continuous EIS scans on 500Ω, 5 KΩ, and 15 KΩ resistors in a two-electrode configuration perturbed with 10 Hz, 100 Hz, 1000 Hz, and 10000 Hz 10 mV amplitude AC excitation voltages. EOC noise was estimated as the root mean squared error (RMSE) values for 100 observations recorded using the 10 KΩ, 100 KΩ and 1 MΩ TIA resistors, with the appropriate sampling frequency for each defined signal input frequency to decrease spectral leakage during FFT, and using the 12-bits default ADC resolution of the Teensy LC. RMS current noise is given by:

$$I_{RMS\;[nA]}=\frac{FFT\;magnitude.VSS}{ADCres.TIAR}$$ (1)

Where *VSS* is the 3.3 VDC voltage source powering the PC, *ADCres* corresponds to the ADC resolution equal to 4096, and *TIAR* refers to the value in Ohms of the TIA resistance (10 KΩ, 100 KΩ or 1 MΩ).

### System validation

TBISTAT was employed for performing EIS on three experimental systems: An AUTOLAB dummy cell circuit composed of a 100 Ω resistor in series with the parallel circuit of a 1*μF* ceramic capacitor and a 1 KΩ resistor; bare thin-film AUIDEs drop casted with 10uL of 10mM K3[Fe(CN)6] in 0.2M KCl solution; ANTI-S100B functionalized thin-film AUIDEs exposed to 316 pg/ml of S100B spiked human plasma samples, and drop casted with 10uL of 10mM K3[Fe(CN)6] in 0.2M KCl as support solution.

Validation was done using two methods: Visual comparison of impedance magnitude and phase responses along the EIS frequency range obtained by TBISAT and an Autolab/M204 benchtop potentiostat/galvanostat (Metrohm®) equipped with an Autolab® FRA32 module controlled by the NOVA 2.11 software, and a T-test statistical comparison between ΔC results at f = 31.6 Hz obtained by TBISAT and the benchtop potentiostat. Capacitance was obtained from EIS scans following the method described by Rodriguez et al. [38]. The statistical analysis was done using Statgraphics Centurion 18. The assumptions of normality, homoscedasticity, and independence of residuals were assessed to establish the statistical validity of the T-test. All statistical tests were considered significant with a p-value lower than 0.05.

### S100B detection using TBISTAT

EIS measurements were performed using the TBISTAT to quantify the S100B protein. A single-factor experimental design with four replicates was made to assess the effect of S100B concentration on the RCT obtained from the EIS of each AUIDEs biosensor platform. The biomarker S100B was tested in three levels set in a logarithmic scale, using concentrations with clinical utility: 31pg/mL (log₁₀ = 1.5), 100 pg/mL (log₁₀ = 2), and 316pg/mL (log₁₀ = 2.5). The ΔRCT was selected as the response variable, and it was defined as the difference between the RCT obtained from EIS for S100B testing (tRCT) and the basal RCT (bRCT) obtained from EIS on anti-S100B/BSA functionalized working surface.

Ten microliters of the spiked human plasma samples were drop casted on the WEs surface of AUIDEs and left to dry at RT. WEs were rinsed with DW after 15 minutes of antigen-antibody binding and left covered at RT. AUIDEs were then connected to the TBISTAT for the electrochemical measurements and tested in a frequency range of 1 to 10,000Hz with a 10 mVp analog excitation signal. Electrochemical measurements were carried out using 10uL of 10mM K3[Fe(CN)6] in 0.2M KCl as a support solution. Variation of RCT was recorded to evaluate changes in impedance after 15 minutes of antigen-antibody binding. Typical semicircular behavior in the range corresponding to high frequencies associated with the electrode redox probe was observed.

A statistical analysis was done using Statgraphics Centurion 19. Initially, the assumptions of normality, homoscedasticity, and independence of residuals were assessed to establish statistical validity graphically and analytically. A one-way Welch ANOVA was applied to check for differences between treatment groups (S100B concentrations), followed by Games-Howell as a post-hoc test. A regression model (calibration curve) was developed together with a lack of fit test to determine model adequacy to the response variable. Model suitability was established considering global model significance, coefficients significance, and analysis of residuals structure. All statistical tests were considered significant with a p-value lower than 0.05.

The limit of detection (LOD) was determined by Eq 2, as follows:

$$LOD=\frac{3.3\;SD}{m}$$ (2)

where SD is the average standard deviation of the response, and m is the calibration sensitivity, determined by the slope of the calibration curve.

## Results

### Fabrication

**Fig 4:**
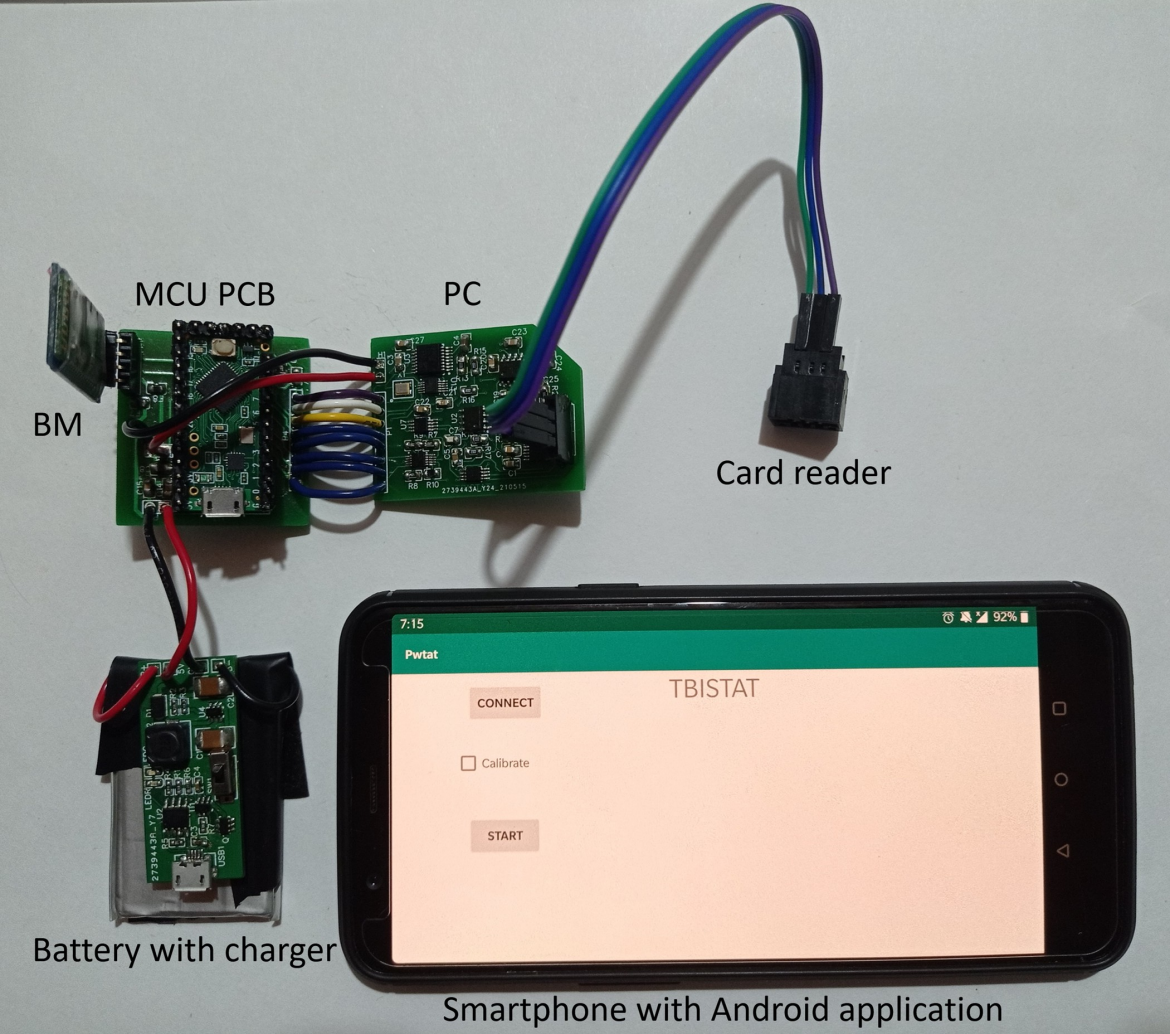
Components of TBISTAT connected for EIS measurement on an AUIDE cell. LiPo battery and boost circuit (bottom left) powers the MCU through two cables (red 5 V, black ground). The BM is connected to the MCU (top left) using four-pin male-female headers. The MCU is connected to the PC through ten cables (two for 3.3 V and ground (red and black), two for I2C communication (SDA purple, SCL white), one for ADC (yellow), and five for Analog switches and TIA resistance multiplexer (blue)). A card reader (top right) is connected to the electrochemical cell female header connector (bottom right of PC). The smartphone is shown running the TBISTAT application.

Fig 4 shows the fabricated hardware modules connected for EIS measurements and the developed Android application running on an Android 7.1 OnePlus 5T smartphone. The hardware occupies 72 *cm*^2^ and 216 *cm*^3^ when extended on a planar surface, as seen in Fig 4. Significant reductions in device volume can be achieved by positioning each PCB on top of each other.

### Power requirements

Table 2 shows the current draw of TBISTAT for continuous use in standby mode and during EIS using a 1000 mAh LiPo battery as energy source. Battery life can last up to 11 hours of continuous EIS scans with a 100 Ω resistor as measured impedance. Higher duration can be achieved using a 2000 mAh LiPo battery, which should last for at least 22 hours of continuous EIS measurements.

**Table 2:** Current draw of TBISTAT for standby and operation modes.

Condition	Current	Battery duration
Standby mode*	60 mA	17
EIS (1Hz to 10KHz) **	90 mA	11

* AD5933 not powered, BM powered, but no scans being made.

** Continuous EIS scans.

### Electrical noise

**Fig 5:**
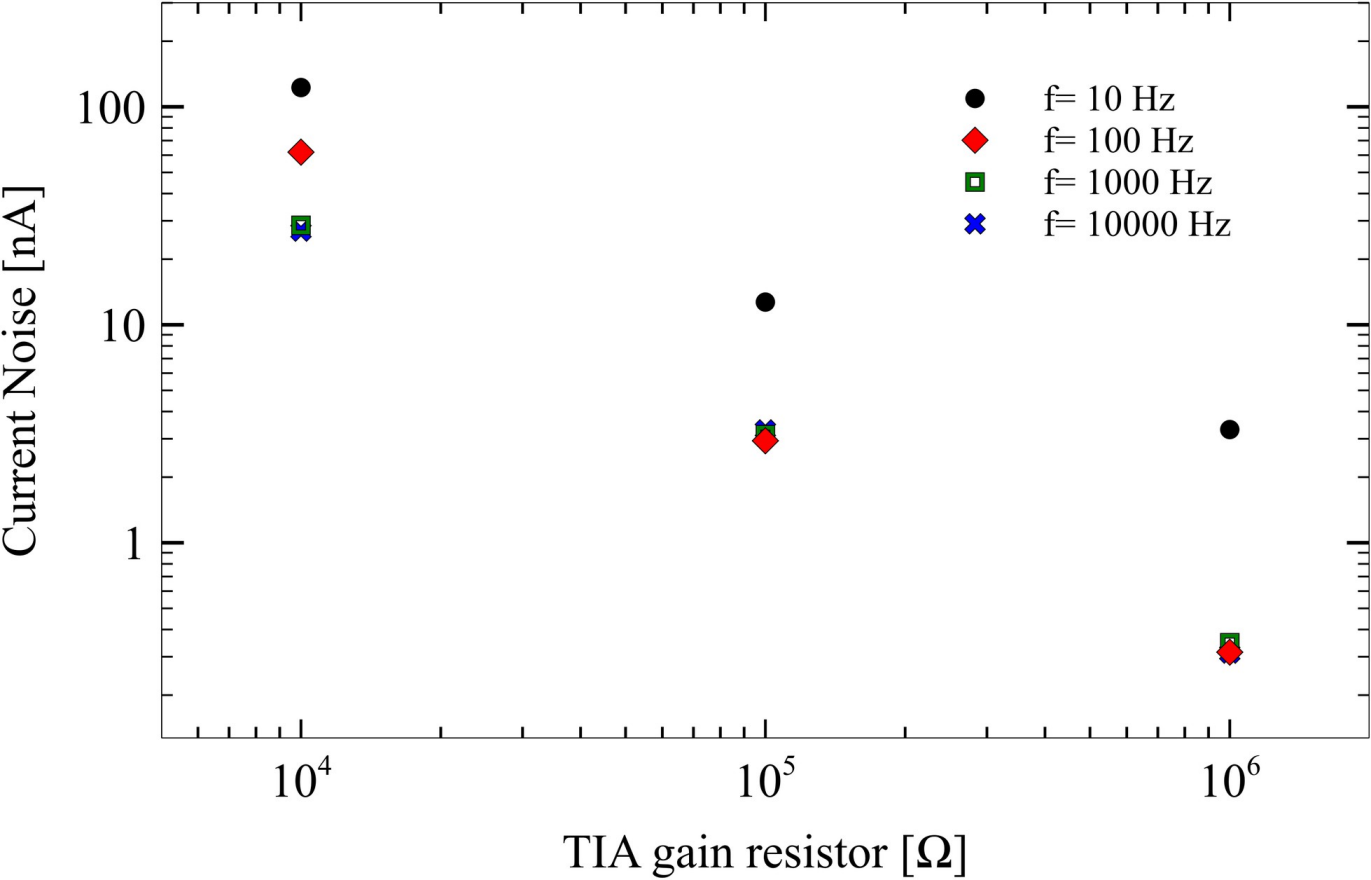
Current noise of TBISTAT for the 10–10000 Hz bandwidth and 10 KΩ-1MΩ TIA gain resistors.

TBISTAT current noise stayed between 0.3 nA and 130 nA for the range of frequencies and impedances used (Fig 5). The noise was higher in low frequency-low impedance EIS scans and remained below 10 nA at higher frequencies and higher TIA resistors values. TBISTAT noise levels were comparable to those reported by Jenkins et al. [22], even though teensy LC has 8 bits less nominal ADC resolution than the 24-bit ADC used by the former. The noise values presented on TBISTAT could be explained by the use of hardware (Sallen-Key) and software filters (five-point median filter) and a highly precise impedance measurement device with low spectral leakage given by accurate frequency resolution definition and a 1000-point FFT.

### System validation and performance

#### Dummy cell experiment

**Fig 6:**
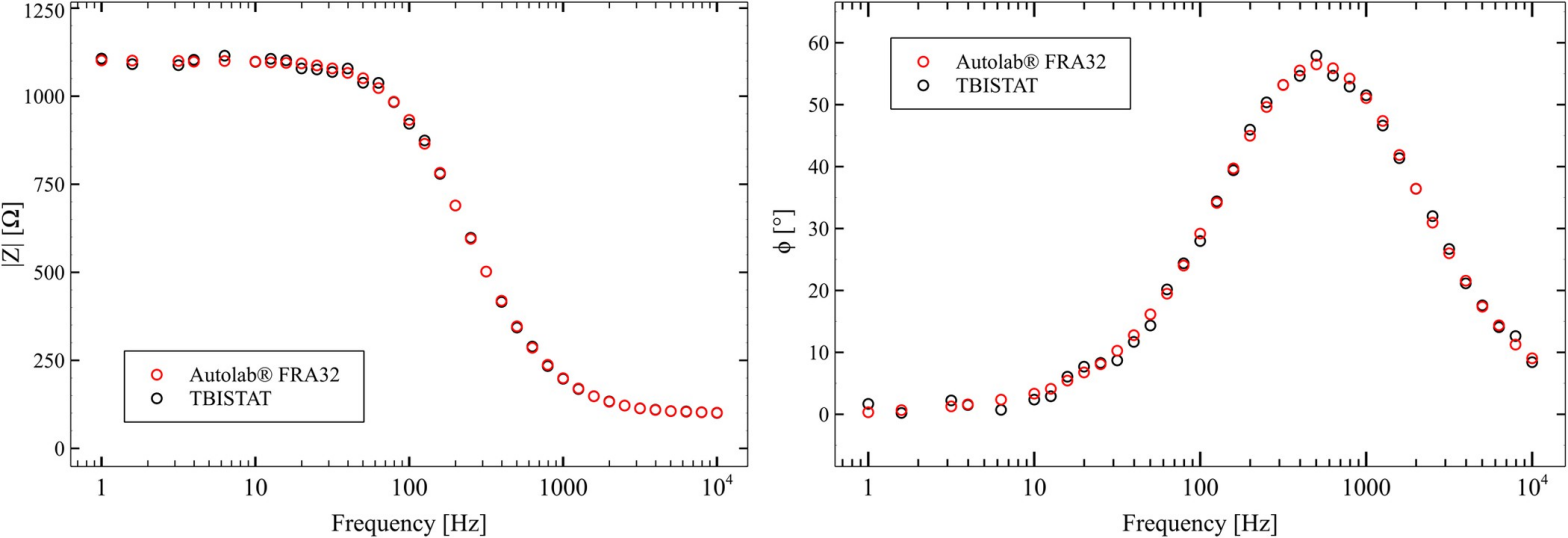
EIS magnitude and phase plots for TBISTAT and reference instrument for dummy cell excitation.

TBISTAT EIS magnitude and phase values were highly similar to those obtained with the reference instrument (Fig 6). However, as expected, higher discrepancies were found at frequencies lower than 10 Hz, which could be explained by higher signal distortion and gain loss due to the high pass filter conditioning AD5933 AC signal generator.

#### Bare AUIDEs drop casted with 10uL of 10mM K3[Fe(CN)6] in 0.2M KCl solution

**Fig 7:**
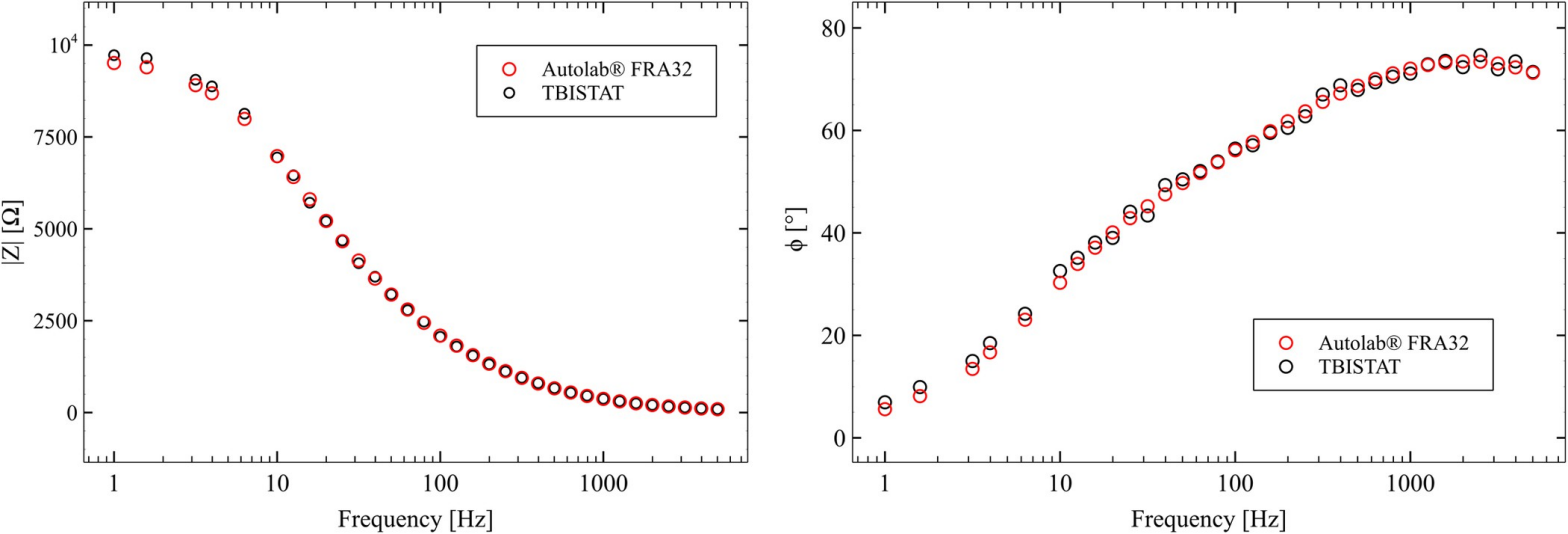
EIS magnitude and phase plots for TBISTAT and reference instrument for measurements in 10mM K3[Fe(CN)6] in 0.2M KCl solution.

EIS scans phase values in the potassium ferricyanide solution using TBISTAT showed higher differences (circa 2°) to those found with the reference instrument (Fig 7). Magnitude values were reasonably close to the reference instrument (less than 2% differences). Higher variations in phase values were found in lower frequencies (less than 10 Hz), which resulted in marked differences with the reference instrument. Nevertheless, the TBISTAT employed calibration strategy successfully prevented discontinuities in impedance measurements when the TIA resistor was increased.

#### ANTI-S100B functionalized thin-film AUIDEs exposed to 316 pg/ml of S100B spiked human plasma samples

**Fig 8:**
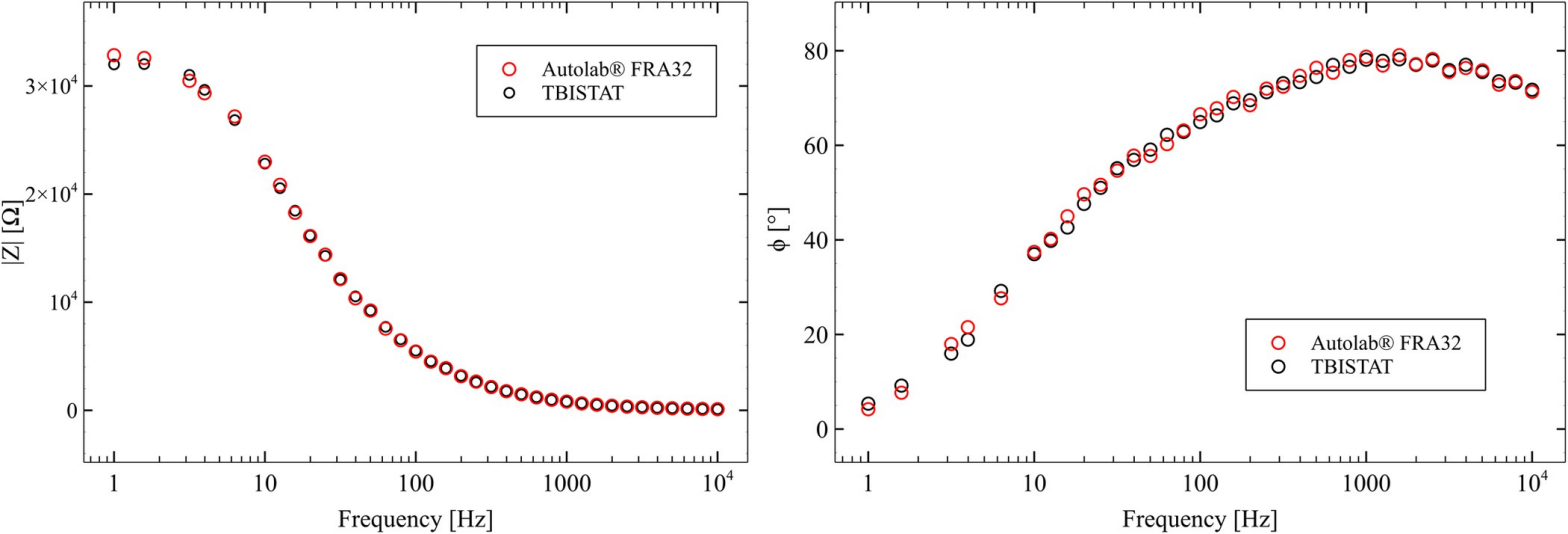
EIS magnitude and phase plots for TBISTAT and reference instrument in ANTI-S100B functionalized thin-film AUIDEs exposed to 316 pg/ml of S100B spiked human plasma samples.

**Fig 9:**
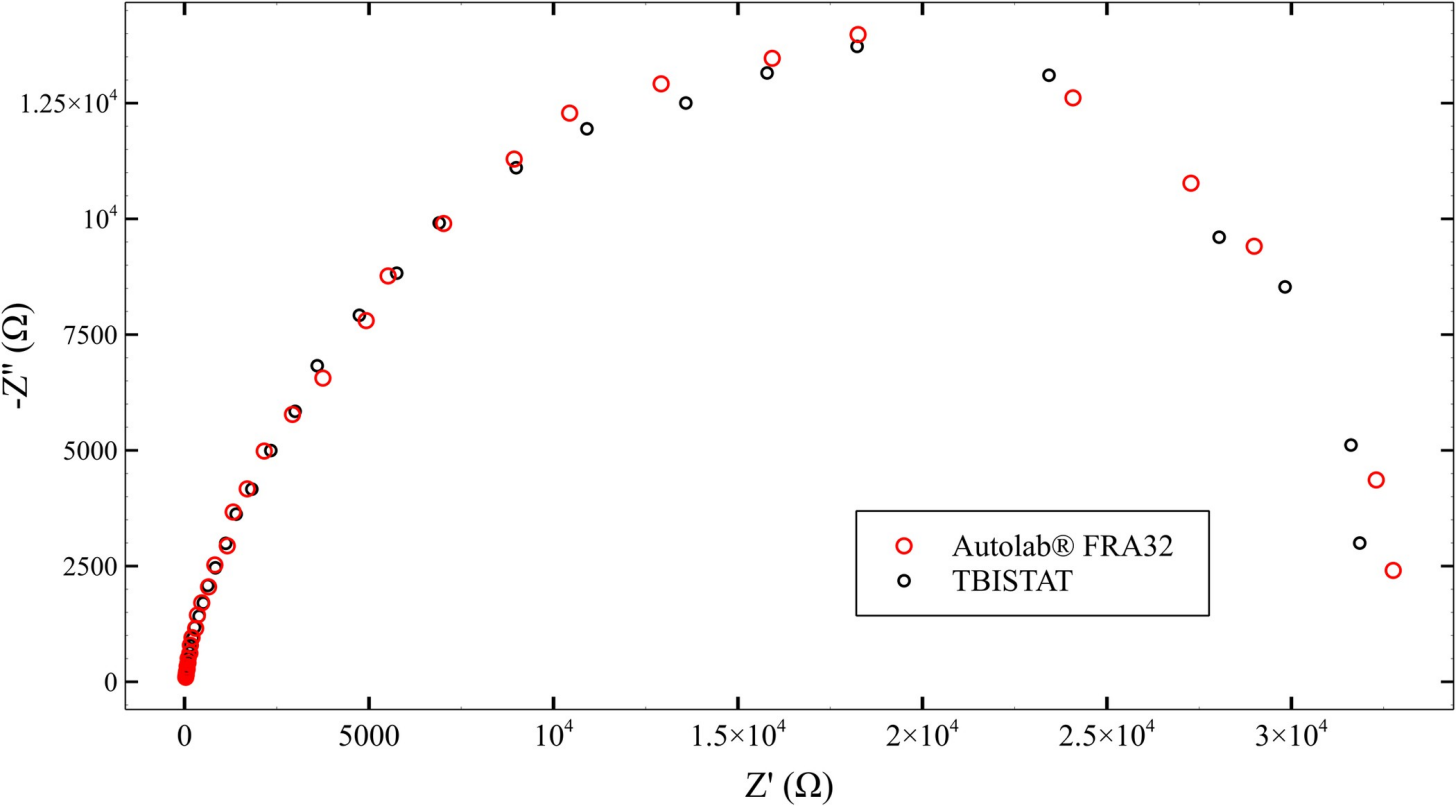
Nyquist plot comparisons of TBISTAT and reference instrument in detecting S100B in an ANTI-S100B functionalized thin-film AUIDEs exposed to 316 pg/ml of S100B spiked human plasma sample.

EIS scans with TBISTAT resulted in similar phase and magnitude values to those obtained with the reference instrument (Fig 8). Higher impedance at lower frequencies showed higher variations with respect to the reference instrument, which resulted in marked differences in real and complex values in the obtained Nyquist plots (Fig 9). The use of a five-point median filter allowed higher precision than expected in the 1–10 Hz frequency range, which was thought to sustain altered phase/magnitude values due to the high amplifier gain and low-current scenario given by the high magnitude impedance. The programmable external clock of the AD5933 prevented excitation signal distortion at frequencies below 1 KHz, and the designed impedance analyzer system maintained sufficient precision in the measurements (less than 2.5% difference in magnitude and phase value vs. reference instrument), which together accounted for a sufficiently accurate EIS capable portable potentiostat. A video showing TBISTAT operation during S100B electrochemical detection using AUIDEs can be found in the S6 File.

A T-test was carried out between the capacitance results obtained with TBISTAT and the reference instrument (Table 3). Both standardized skewness and kurtosis values were within the (-2,2) range, so samples could be considered to come from normal distributions. No significant differences were found in the sample variances using Levene’s test, and no structure was found in the residual plot. Considering that the T-test p-value was higher than 0.05, the null hypothesis could not be rejected. Thus, there was no statistically significant difference between the means of the two samples. Additional information regarding the results of the statistical analysis for potentiostat validation can be found in S7 File.

**Table 3:** Statistical analysis of single-frequency analysis experiment.

	Relative change in capacitance (%) of Au/Cys/Ab/BSA/316 pg/ml AUIDEs	
Experimental run	BENCHTOP	TBISTAT
**1**	49.17264105	45.39904809
**2**	56.02429244	54.42103458
**3**	51.60599865	48.04601753
**4**	55.83877576	56.6168629
**5**	48.88026492	54.80168404
**Mean**	52.30439456	51.85692943
**Standard deviation**	52.93074526	53.1485057
**Skewness**	0.221046	-0.581177
**Kurtosis**	-1.38102	-0.991419
	**Test**	**P-Value**
**Levene’s**	1.81495	0.2148
**T-test** *H*_0_: *μ*_1_ = *μ*_2_, *α* = 0.05	0.167647	0.871021

### S100B detection using TBISTAT

EIS measurements of S100B were performed in spiked human plasma samples to evaluate a possible future application of these biosensors in medical diagnosis. The results obtained from the EIS of the biosensor in the presence of 10mM K₃[Fe(CN)₆] redox probe for the quantification of S100B were assessed, exhibiting non-homogeneity of variance. A normal distribution for the response variable (ΔRCT) was observed and residual independence. The analysis of variance (Welch-ANOVA) and the post hoc analysis showed statistically significant differences in the ΔRCT signal between each tested concentration (p < 0.05). Information regarding experimental runs and statistical analysis are found in S7 File.

**Fig 10:**
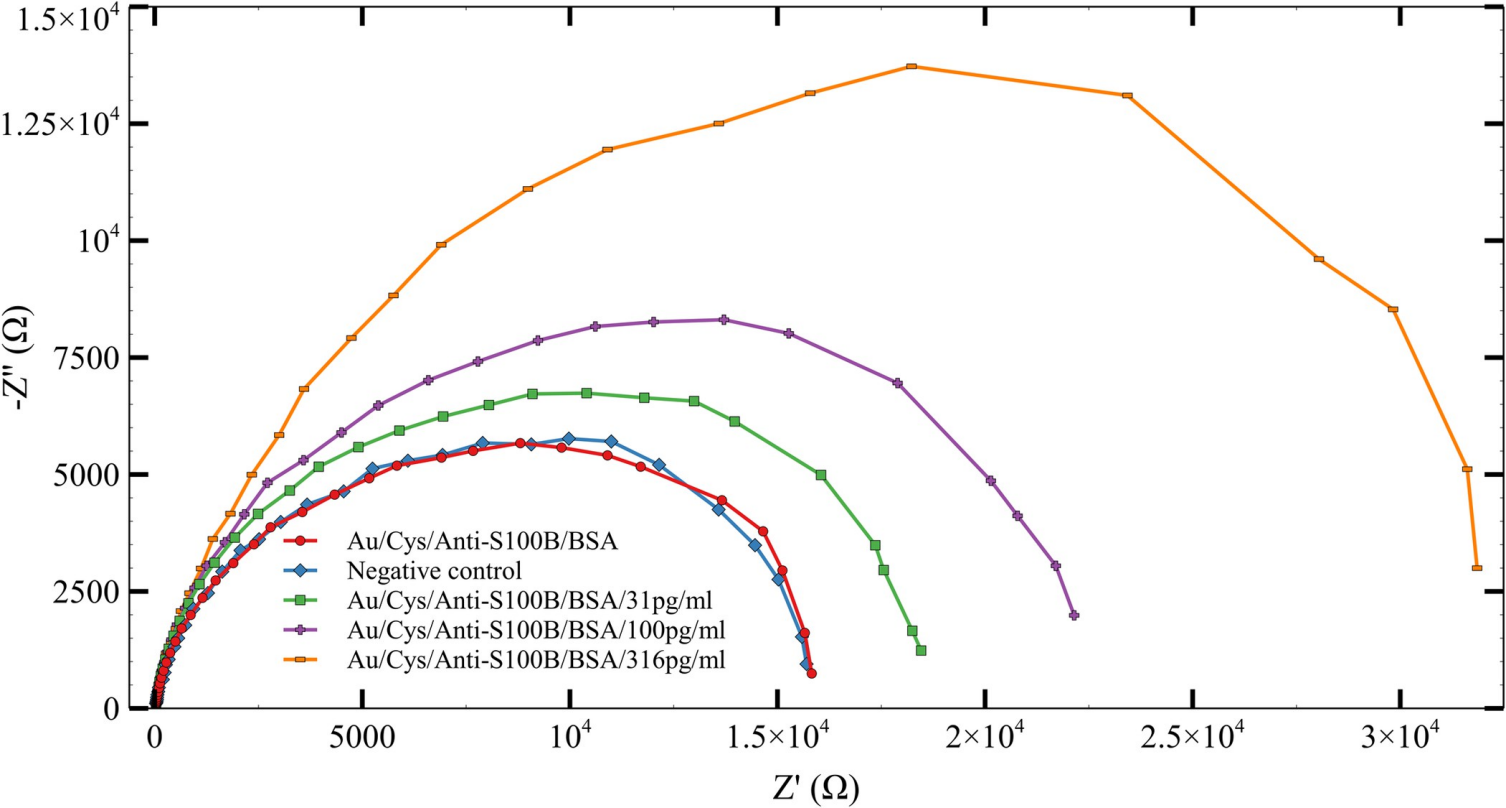
Nyquist plots of AUIDEs experimental results in spiked human plasma samples for the quantification of S100B in the 31–316 pg/mL range.

Fig 10 shows the EIS spectra obtained for the quantification of S100B in the range of detection. The basal signal corresponds to the AUIDE/Cys/anti-S100B/BSA without plasma addition, while negative control refers to plasma without S100B. A proportional increment was observed in the ΔRCT with the successive increments of the S100B concentration. The response of electrodes (ΔRCT) was consistent and showed a maximum relative standard deviation (RSD) of 19.15%, indicating good reproducibility of the S100B detection.

**Fig 11:**
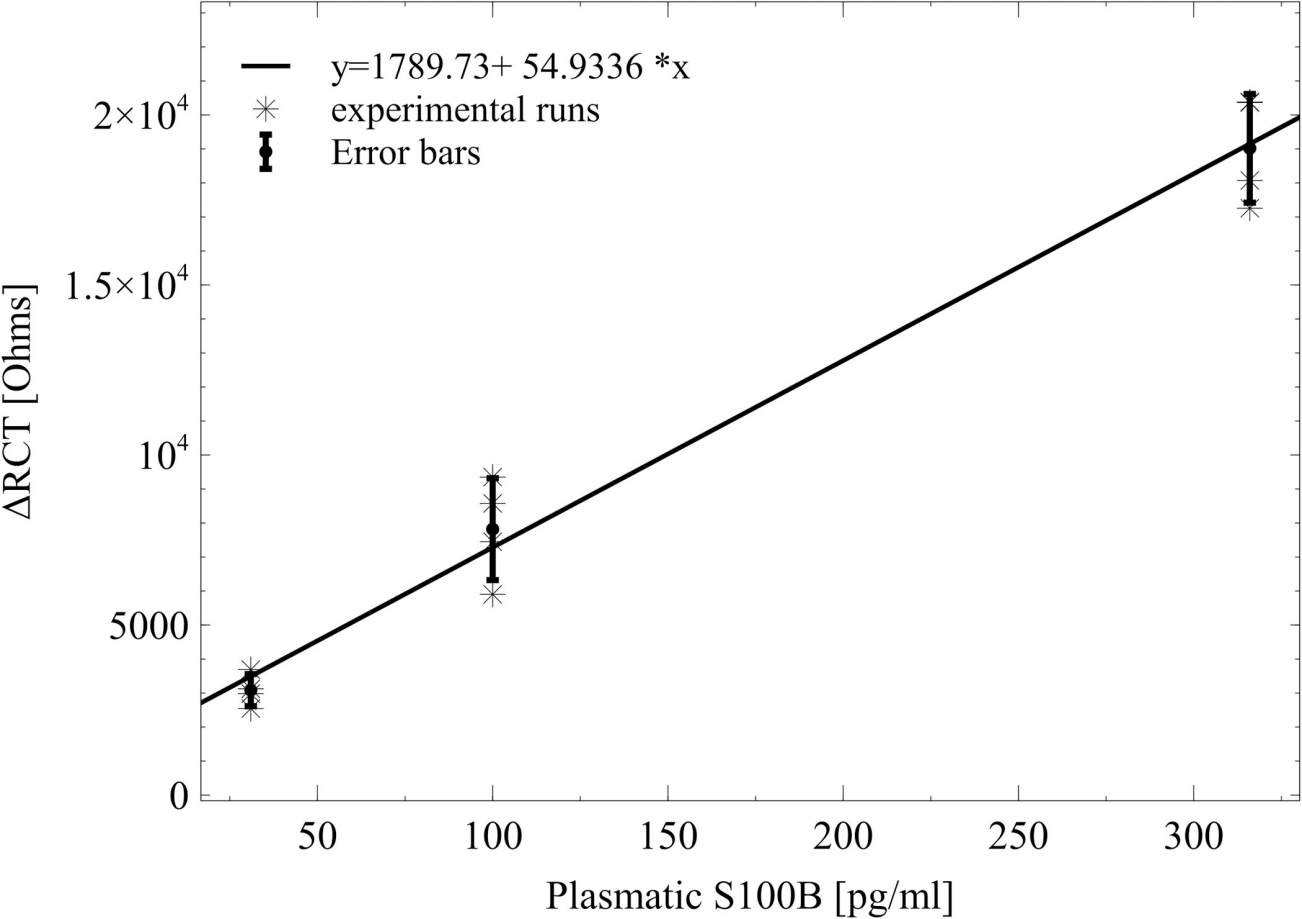
Calibration curve for S100B in spiked human plasma using AUIDEs. y = ΔRCT; x = [S100B] (pg/mL).

The response of the platform in the linear detection range between 31 and 316 pg/mL was modeled by the regression equation y = 1789.73+ 54.9336 * x (n = 4), where x is the concentration of S100B (pg/mL) in real scale and y the change in RCT (ΔRCT) measured in Ohms (Fig 11). Each point on the calibration curve represents each independent measurement, and the error bar represents the standard error of the mean. The model was found suitable for the experimental results since its coefficients were significant, no structure was found on its residuals, and the lack of fit test was not significant. The LOD obtained for AUIDEs was 35.73 pg/mL. The statistical test results are found in S7 File.

### Brain injury support information

**Fig 12:**
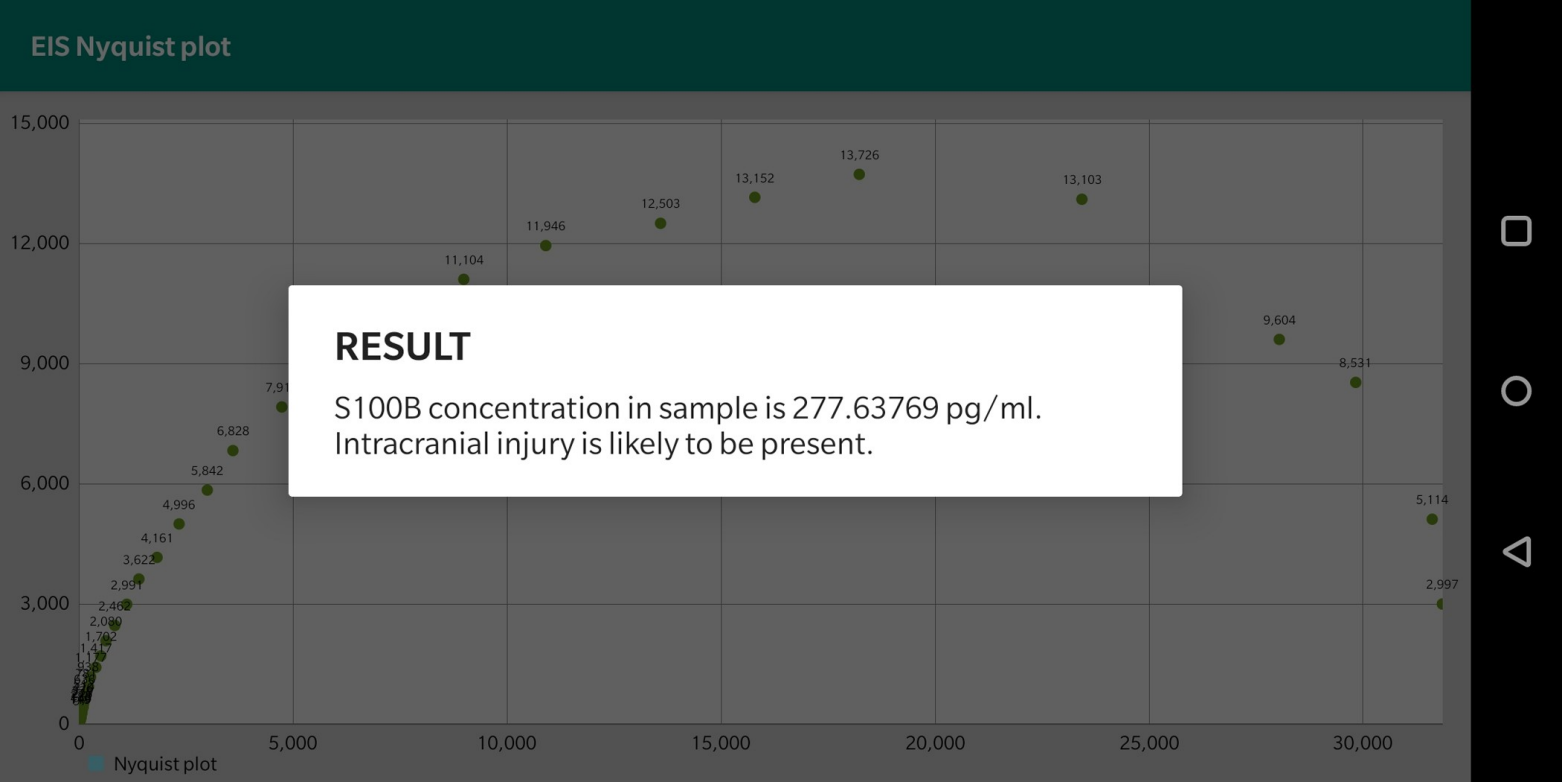
Brain injury support information after the EIS test.

TBISTAT takes ΔRCT values from the EIS scan for finding S100B concentration in AUIDEs (Fig 12). Brain injury support information is shown as a message that suggests the presence or absence of a TBI depending on the measured S100B concentration in the blood sample.

## Discussion

This work has developed an open-source EIS-capable portable potentiostat controlled by a wirelessly interfaced Android application that detects and quantifies the concentration of the S100B TBI biomarker from plasma samples in clinically relevant conditions. Previous work from Jenkins et al. [22], Ainla et al. [24], and Pruna et al. [26] has laid a solid foundation in the understanding of the intricacies and possibilities in terms of building a portable, wireless potentiostat prototype that is affordable and easy to adapt to a wide range of applications concerned with the detection of an analyte of interest.

Apart from Jenkins ABE-Stat, to our knowledge, the TBISTAT is the only other portable, wireless, open-source potentiostat capable of conducting EIS measurements. The TBISTAT circuit components and PCB fabrication can be acquired for less than 80 USD for one unit, comparable to the 105 USD cost of Jenkins ABE-Stat. Circuit components suppliers and packaging have been carefully chosen to allow fast acquisition and easy PCB assembly using standard soldering techniques with a hot iron and a soldering rework station. In addition, the circuit schematics, Gerber files, BOMs, operation videos, calibration files, MCU firmware, and Android application project have been made freely available for download without demanding any usage permits. Software and hardware development interfaces such as Arduino IDE, Android Studio, and EasyEDA allow rapid setup, circumventing the need for costly licenses and allowing complete access to every aspect of the prototype design and fabrication.

Even though the TBISTAT was designed to detect S100B, several improvements of previous works have been attained to provide more robust and precise impedance measurements. While the TBISTAT was not designed to be a general-purpose potentiostat, configured to perform voltammetric assays and user-defined DC-biased EIS scans as Jenkins et al. ABE-Stat, its performance has been increased in the 1-10KHz, 100–35 KΩ ranges when compared to the former. The Teensy LC MCU employed in the TBISTAT possesses a higher number of GPIOs than Jenkins ABE-Stat ESP8266. In addition, the Teensy LC true 12-bit ADC allows interrupt-driven ADC with sampling rates as high as 200 KHz, thus avoiding the use of leakage-prone AD5933 single-point DFT [37]. Jenkins et al. employed a 24-point DFT for excitation frequencies below 60 HZ using a DAC software-programmed AC excitation signal, a variation which was thought to result in significant discontinuities in impedance measurements at 2 Hz, 60 Hz, and 2 kHz using ABE-Stat, especially when the current signal was not a perfect sinusoid. The TBISTAT improves the design of Jenkin’s impedance analyzer by utilizing its own impedance analyzer circuit and a customized 1000-points FFT programmed in the Android application. The accurate sampling frequency definition in the Android application of the TBISTAT allows a fully customizable FFT frequency resolution for logarithmically spaced AC excitation signal frequency points, significantly reducing FFT spectral leakage and avoiding the use of windowing techniques before or after FFT. Furthermore, impedance measurement discontinuities when the TIA resistor changes as impedance increases are reduced using the same calibration resistors at the frequency range limits. In addition, the presence of a fourth-pole Sallen key LPF reduces the amplitude and duration of the perturbation, which in turn decreases the overall impact of this event on the electrochemical equilibrium of the AUIDE cell.

S100B detection using TBISTAT and AUIDEs biosensor platform exhibited an acceptable global performance in terms of stability and reproducibility. Given the higher steric hindrance due to the anti-S100B and S100B protein interaction as S100B concentration increased and the electrostatic repulsive forces between the S100B and negatively charged redox species in support solution (10mM K3[Fe(CN)6] in 0.2M KCl) [39], a proportional increment was consistently observed in the RCT to the successive increments in S100B concentration.

The use of AUIDEs with portable potentiostat such as TBISTAT offers various advantages to facilitate further industrial development and commercialization of POCT devices. First, a simple cleaning protocol (RCA-1) before functionalization is feasible for AUIDEs, avoiding expensive reagents and considerably reducing the time required. Secondly, AUIDEs possess a small planar detection area, which allows smaller cell volumes, thus reducing the quantity of antibody solution needed for biosensor functionalization without a negative impact on the biosensor performance. In third place, AUIDEs electrodes do not require a reference electrode for analyte detection during impedance measurements, making the further development of a POC system easier, with fewer operational amplifiers needed in the instrument.

Even though other studies report a wider range of detection and lower LODs for S100B [40–42], this work demonstrates an effective detection of S100B in a clinically relevant range since plasmatic S100B concentrations lower than a cutoff of 100pg/mL [9] ruled out the presence of bleeding in the CT, while levels below 30pg/mL ruled out BBB disruption [10]. Furthermore, many of the sensors found in the literature exhibit limitations for a fast analysis due to the requirement of sandwich-type immunoassays and fluorescent labels. Label-free and simple functionalization chemistry are valuable features of our biosensor, which provides a promising alternative for the fast analysis of biomarkers, even for very small sample volumes.

One simple but important contribution of TBISTAT is the methods it uses to quantify S100B concentration from EIS scans in the AUIDEs biosensor platform. After EIS is done, S100B concentration is calculated using the RCT obtained as the intercept at y = 0 of a three-point regression model from the lower frequency EIS scan, which is then used as input for calculating ΔRCT and S100B with the regression model. In this way, no electrochemical circle fit is needed, and the S100B concentration estimate defines TBI support information for patient diagnosis and treatment.

### Limitations

Various limitations should be described for users interested in using the proposed prototype for their specific purposes. Even though S100B detection using AUIDEs did not require changing DC bias above zero volts, other applications might require such adjustments. A fully programmable DC bias voltage potentiostat using the Teensy LC 12-bits DAC together with a fourth-pole Sallen-Key LPF such as the one designed by Ainla et al. [24] for the UWED is currently being developed.

The TBISTAT can only be used in the 1Hz-10KHz frequency range to measure impedances between 100Ω and 35KΩ. Replacing the Teensy LC 48 MHz cortex-M0+ with a 96 MHz cortex-M4 Teensy 3.2 could increase sampling frequency and allow the ADC of higher frequency signals without incurring any PCB changes. In addition, a 100 KHz Sallen-key LPF could replace the 10KHz already found on TBISTAT. Higher impedances can be measured by using the 10 MΩ TIA resistor already found on TBISTAT.

The TBISTAT employs a simple voltage divider circuit for VGV and uses 0.1% tolerance resistors to achieve a low (less than 5 mV) DC offset between reference and working electrodes. This value reduces the usable AC voltage span for ADC to less than 500mV, as it induces DC currents in the TIA in the lower impedance ranges of each TIA gain resistor setting which change VGV to less than 3.3/2 V. In addition, this circuit is dependent on device voltage supply, which can have slight variations following LiPo battery discharge. The addition of a 3.3 V high precision analog voltage reference using an ISL60002 IC and a 3.3 V Rail splitter for analog reference such as TL2426 IC [22] could be used as an alternative to the VGV circuits employed in the proposed design to allow a more precise reference voltage for zero DC bias EIS measurements.

Further studies are needed to identify nuisance factors affecting the electrochemical measurements using TBISTAT, such as temperature, humidity, electrical noise, and tests in real settings beyond the laboratory. Experiments should be carried out at clinical facilities with TBI patient’s blood for both the biosensor platform and the developed potentiostat at a clinically relevant range of concentrations of S100B. Moreover, the inability to measure S100B directly in plasma samples without the need for additional redox probes should be circumvented in future iterations of the developed biosensor platform so that electrochemical detection can be made in a non-faradaic manner. More detailed studies are now underway to implement non-faradaic electrochemical measurements using AUIDEs since the use of a redox solution could represent a limit for scaling the technology to a marketable stage.

Finally, machine learning algorithms for EIS analysis could support TBI diagnosis and treatment by using supervised learning in EIS Nyquist plot analysis. This route could pose an alternate solution to employing an electrochemical circle fit algorithm to find RCT value and eventually S100B concentration and its correlation to TBI outcomes.

## Conclusions

We have described the development and validation of a portable, wireless, open-source potentiostat capable of performing EIS on AUIDEs to detect and quantify S100B in plasma at clinically relevant concentrations. The TBISTAT occupies 216 *cm*^3^, weighs 120 g, and has an approximate manufacturing cost of 80 USD. Its design is built upon the potentiostats made by Jenkins et al. [22] and Ainla et al. [24], improving accuracy in phase and magnitude measurements and reducing discontinuities in the overall impedance calculations along the 1-10KHz frequency excitation range and between 100Ω and 35KΩ. Source code for MCU firmware and Android application, Gerber files, schematics, and device operation video of TBISTAT have been made freely available for download to promote its use, enhancement, and employment in applications in either medical, animal, food or agroindustry. Furthermore, the modularity of the design allows easier component changes according to the application demands in power, frequency excitation ranges, wireless communication protocol, signal amplification and transduction, precision, and sampling frequency of ADC, among others. In addition, the use of minimal, easy acquirable open-source hardware and software, together with high-level filtering, low-cost, accurate ADC, wireless communication, and the simple user interface, provides a framework for facilitating EIS analysis for similar POC applications such as the one presented in this work and for developing affordable diagnostics and POC biosensors integrated systems. Improvements to the prototype, such as adding a high precision analog voltage reference and a 3.3 V Rail splitter for analog reference, could increase the device capabilities and range of possible applications to meet user-specific demands.

## Supporting information

S1 File Hardware considerations. Additional details concerning hardware design, circuit schematics and PCB design.
(DOCX)

S2 File Software design. Additional details concerning software design.
(DOCX)

S3 File MCU firmware and Android application files. (RAR)

S4 File BOMs, circuit schematics and Gerber files. (RAR)

S5 File TBISTAT calibration file. (XLS)

S6 File Video file. (MP4)

S7 File Statistical tests and figures. (DOCX)

S8 File Configuration values for AC excitation signal and ADC. (XLSX)
