# Supplementary material for: TBISTAT: An open-source, wireless portable, electrochemical impedance spectroscopy capable potentiostat for the point-of-care detection of S100B in plasma samples
Source: PLoS One. 2022 Feb 7;17(2):e0263738. doi: 10.1371/journal.pone.0263738 (PMC8820642; doi:10.1371/journal.pone.0263738)
Supplement: S1 File — Additional details concerning hardware design, circuit schematics and PCB design. (DOCX) [file pone.0263738.s001.docx]

**S1. Hardware design**

**LiPo battery boost, charger, protection circuit module**

Figure A shows the circuit schematic of the LiPo Battery boost/USB charger module board. Briefly, the circuit consists of a micro-USB 2.0 female connector connected to a TP4056 IC, which is configured as a 4.2 V, 580 mA constant-current/constant-voltage linear charger for a single cell LiPo battery. FS312F-G is an IC configured to protect the LiPo battery from over-discharge (2.9 V limit), overcharge (4.25 V limit), and overcurrent (150 mV detection voltage). An MT3608 is used as a step-up converter (boost) circuit to increase the 3.7 V of the LiPo battery to 5.0 V for MCU and BM voltage supply. The MT3608 also includes under-voltage lockout, current limiting, and thermal overload protection to prevent damage in the event of an output overload.


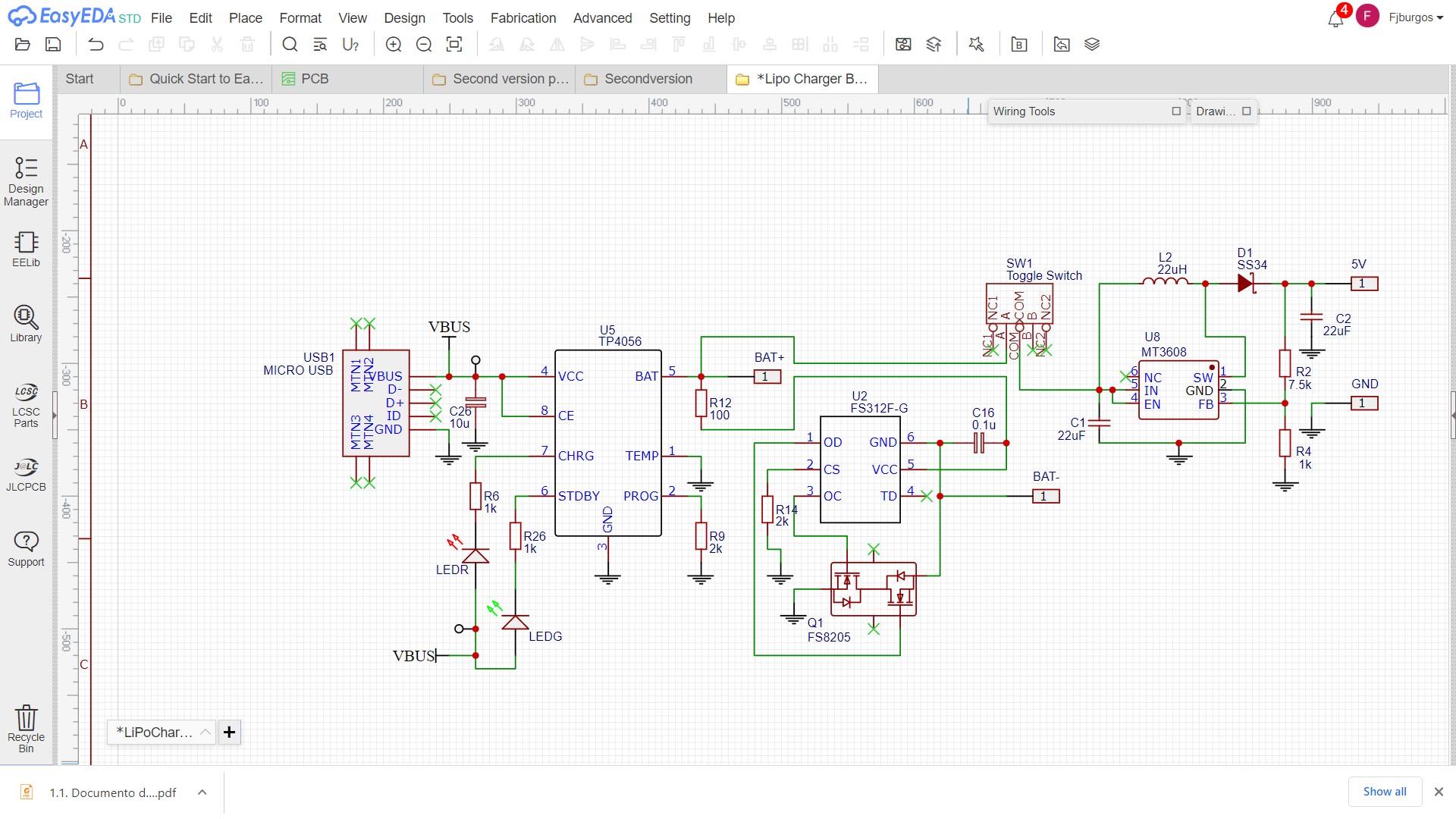


**Fig A**. Circuit schematic of LiPo Battery boost/USB charger module board. Credits to <https://www.youtube.com/watch?v=Fj0XuYiE7HU&t=1s&ab_channel=GreatScott%21>

**Wireless communication with Bluetooth module**

The TBISTAT communicates with a smartphone or a tablet using either the wireless BLE protocol or B2.0. A host program in the smartphone receives the input parameters of the experiment from the user, communicates the experimental protocol to the TBISTAT, receives the raw data (ADC) as the result of the experiment from the TBISTAT, and visualizes the data for the user in a real-time Nyquist plot. The modules HC06 and HM-10 were used for performing wireless communication between TBISTAT and a smartphone. HC06 is a 5V Bluetooth module designed for establishing short-range wireless data communication between MCUs, and other embedded systems acting as slave devices, with transmission speeds up to 2.1Mb/s. HC06 is one of the cheapest modules for wireless data transmission. It also adds flexibility to the design as it can easily be connected, only requiring four I/O pin connections (VCC, ground, Tx, and Rx). It uses the frequency hopping spread spectrum technique (FHSS) to avoid interference with other devices and to have full-duplex transmission. The device works on the frequency range from 2.402 GHz to 2.480GHz. HM-10 is a low Energy Bluetooth 4.0 communication module with an equivalent four I/O pin connections to the HC06. It uses the same 2.4 GHz radiofrequency as HC06 and enables the BLE protocol for very low power applications, providing more uptime before LiPo battery discharge.

Communication between BMs and MCU is done using the universal asynchronous receiver/transmitter (UART) interface, which is a block of circuitry responsible for implementing serial communication using two channels, one for transmission (Tx) and one for reception (Rx). Both modules permit serial transfer as high as 115200 Bauds.

**MCU**

Figure B shows the circuit schematic of the MCU module board. It consists of a teensy LC MCU with defined circuits for power, I2C communication, analog switch ICs control, and serial communication. It has four header modules: One for 5.0 V input voltage and ground, one for 3.3 V output voltage and ground, a four-pin header for HC06/HM-10 connection, and an eight-pin header for I2C serial data (SDA), I2C serial clock (SCL), three multiplexer control signals for transimpedance amplifier resistance change (A00, A01, and EN), two analog switch control signals for DDS power (ADON) and electrode connection (EIS), and one for EOC acquisition for ADC.


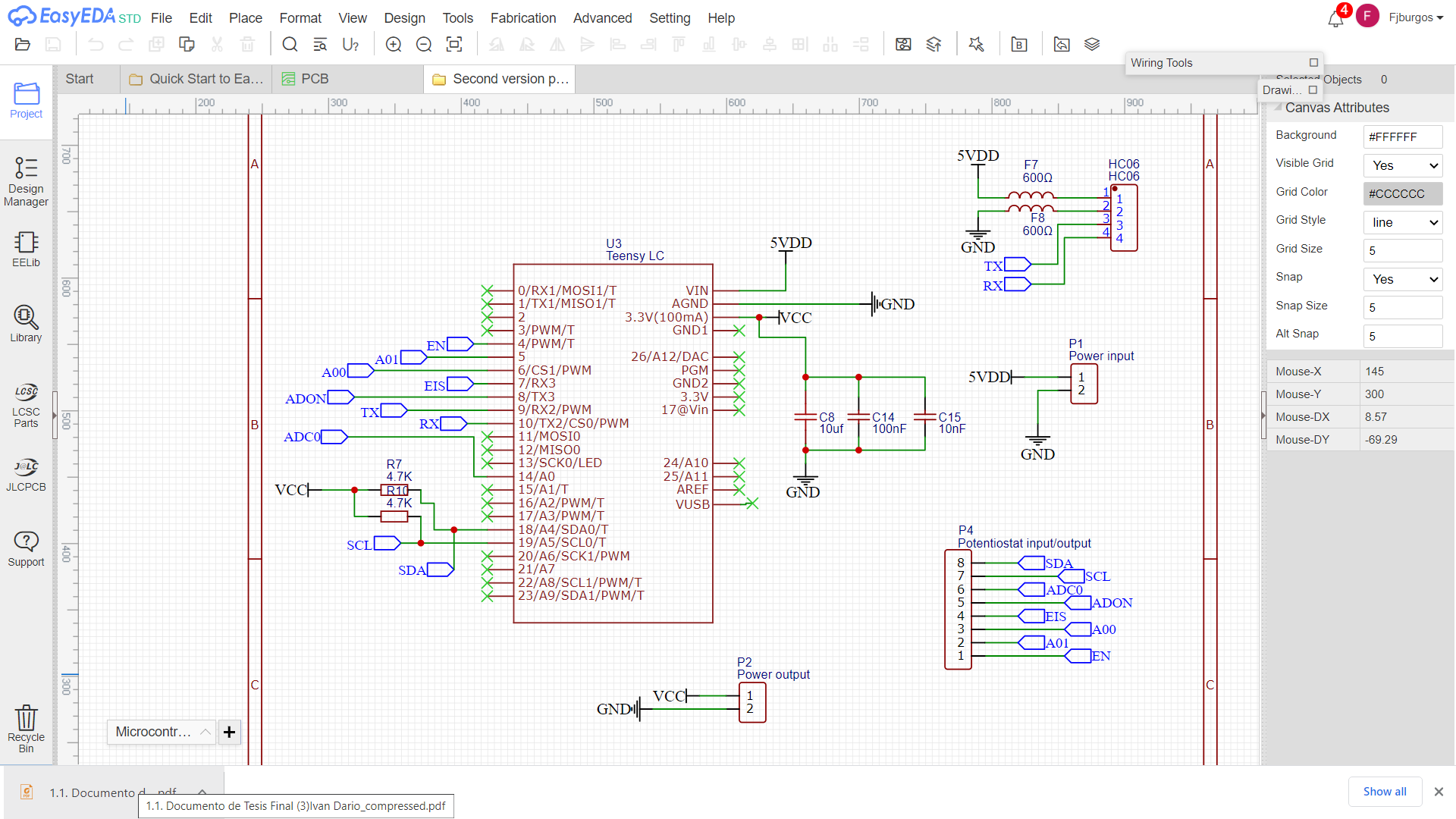


**Fig B**. Circuit schematic of MCU module board. Original design.

**Digital-analog front-end potentiostat circuit**

Figure C shows the circuit schematic of the PC module board. It comprises three modules: The AC excitation signal design module made up of the clock generator IC, the DDS, and the signal conditioning circuit; The PC module made up of analog switch ICs, TIA circuit, and signal filtering circuit; and the mixed-signal grounding module. Noise reduction and mixed-signal grounding were achieved by using ferrite beads for both ground and power supplies of MCU, PC, and BM. In addition, only one connection point between analog and digital grounds was defined to suppress high-frequency noise from the ADC and DDS excitation signal. The electrochemical cell connector was set for three-electrode platforms. Hence, counter and reference ports must be manually interconnected for performing EIS in two-electrode platforms.


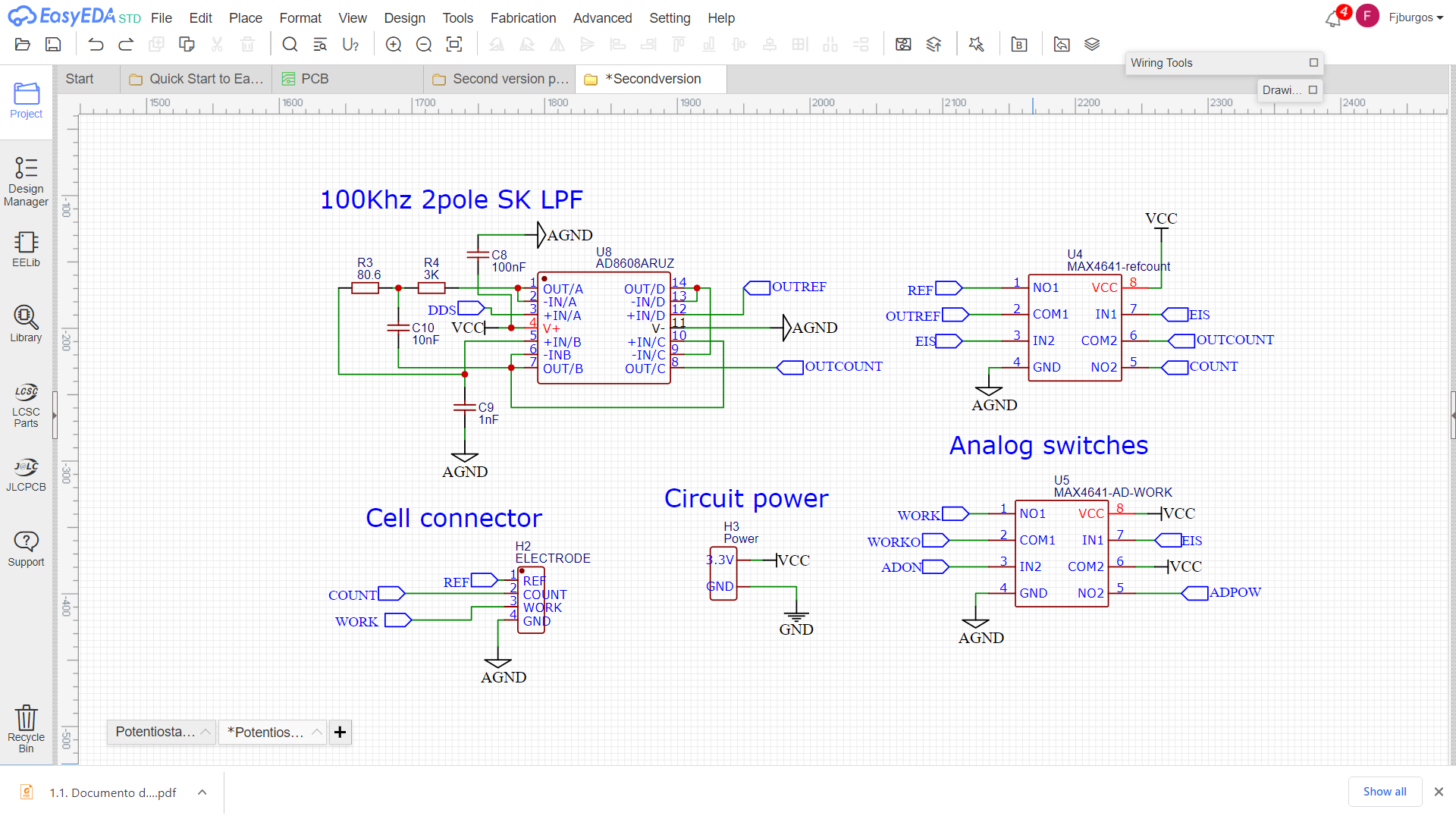


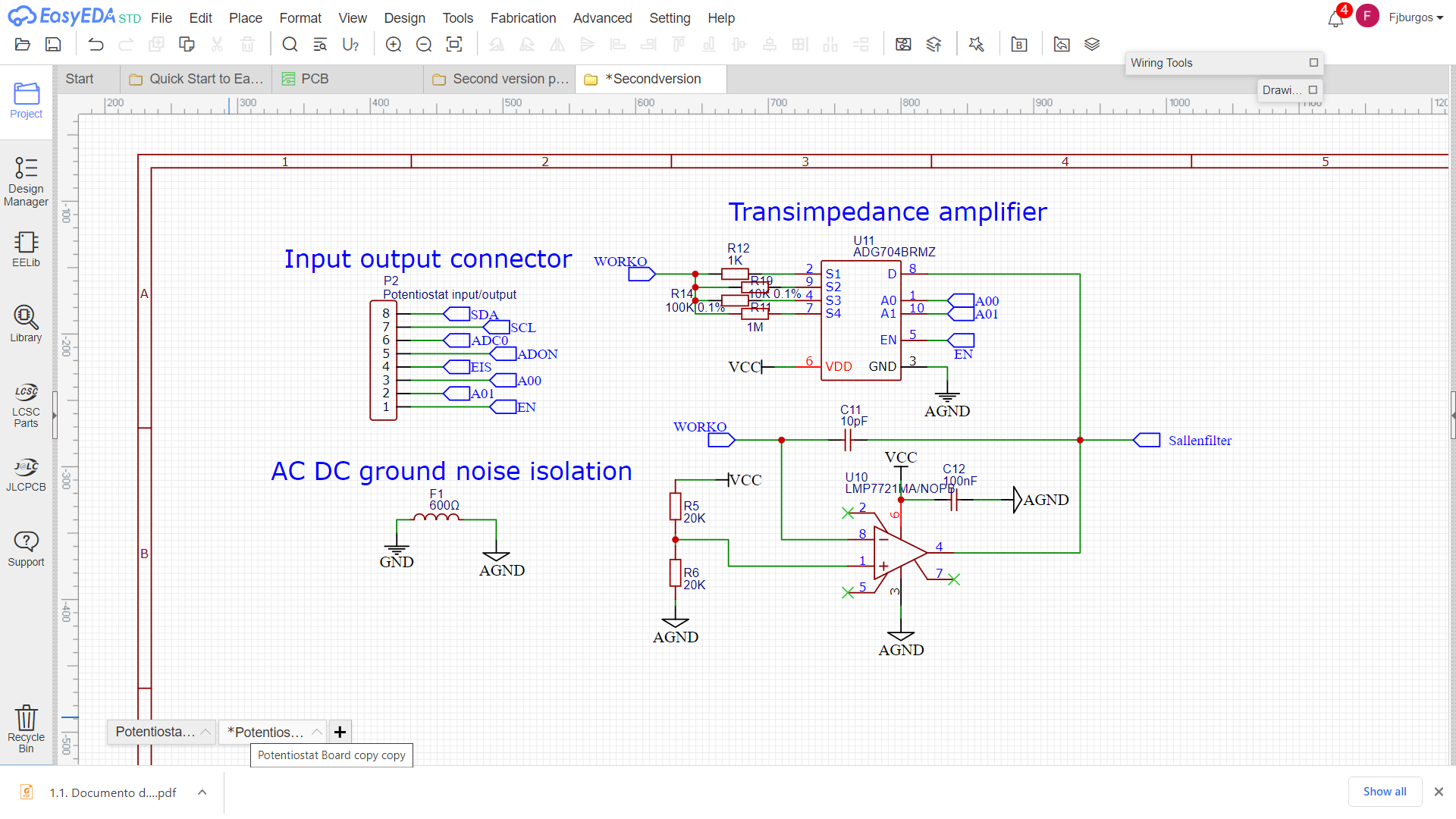


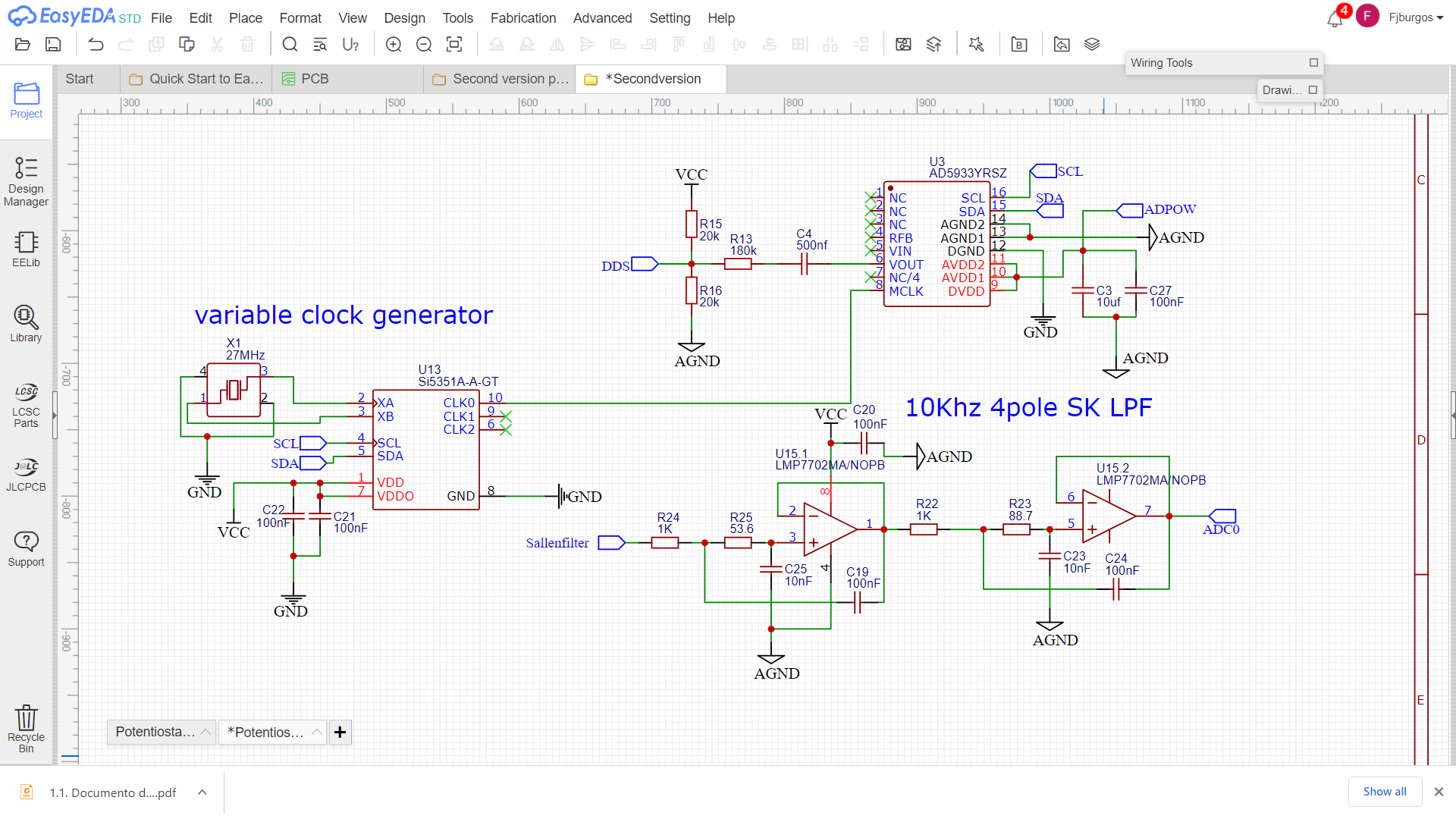


**Fig C**. Circuit schematic of PC module board. Original design.

**AC excitation signal design module**

The generation of an AC signal from DC voltage sources relies on the adequate use of a DDS IC. Similar to previous studies [1,2], The TBISTAT employs the AD5933 network analyzer IC, which consists of a 27-bit direct digital synthesis (DDS) sine excitation voltage generator, a digital-to-analog converter (DAC), and a programmable gain amplifier (PGA1) which determines the AC signal amplitude in four possible ranges [3].

**Ac excitation signal construction and impedance measurements**

According to the datasheets of the AD5933 and UG-364 evaluation board [4,5], the AD5933 can produce 1KHz-100KHz AC signals and do impedance measurements within the same frequency range without any external components. However, as explained by [6], the lower limit of this frequency range is affected by the ADC sampling rate, which in turn defines the resolution of the DFT as shown by the following equations:

| $ADC sampling rate=\frac{MCLK}{16}$ | (1) |
| --- | --- |

| $DFT resolution=\frac{ADC sampling rate}{1024}$ | (2) |
| --- | --- |

Where MCLK corresponds to the AD5933 internal clock frequency equal to 16.778 MHz. Hence, when using the internal chip oscillator, the AC excitation frequency cannot go below 1 kHz since a 1.04 MHz sampling rate will be imposed by it, limiting the DFT resolution to 1 kHz [6]. Suppose the AD5933 tries to examine excitation frequencies below 1 kHz. In that case, the errors introduced by spectral leakage become very significant and result in erroneous impedance readings since each frequency bin of the DFT will represent 1 KHz frequency increments [7,8]. For the excitation signal frequency to go below 1 kHz, the clock that drives the AD5933 must be changed for each range of frequencies to obtain appropriate DFT resolutions. In this work, the S5351A IC is used as a clock generator connected to the external clock pin on the AD5933, which allows an excitation bandwidth increase between 1 Hz - 100Khz. Using a 27 MHz crystal, the SI5351A sends a square wave signal to the MCLK pin of the AD5933, so that sampling rates can be reduced to appropriate values for each frequency of the AC excitation signal to be generated.

During EIS, electrochemical systems are commonly perturbed with AC excitation voltages of about 5 to 10 mVp to preserve the linear behavior of the EOC [9]. However, potentials as small as 2 mVp and as large as 20 mVp have been employed [10]. The AD5933 can produce AC signals with four preprogrammed AC amplitudes which can be customized with rail-to-rail operational amplifiers by adding an amplifier resistor in the signal conditioning circuit. Even though the AD5933 can provide precise excitation signals at different frequencies with external clock input, this excitation signal has a DC-bias voltage that is different for each excitation amplitude. A DC voltage difference across an electrochemical cell under test might cause polarization which could significantly alter the impedance response of the system by inducing a DC component in the EOC [3]. Therefore, a high pass filter is usually necessary to cancel the DC component of the excitation signal before reaching the unknown impedance. In this work, a high pass filter/amplitude reduction circuit was designed to achieve a 10 mVp, 3.3/2 VDC, biased excitation signal from a 0.1915 Vp, 0.31 V, 1 KΩ DC corresponding to range three of the four, preprogrammed AC amplitudes in the AD5933 and found on the pin 6 VOUT (see Figure C, direct digital synthesizer circuit). The designed high pass filter circuit also induces a signal DC bias of 3.3/2 to provide a reference DC voltage via a simple voltage divider circuit that uses two low-tolerance (<0.1%) 20 KΩ resistors, also known as virtual ground voltage (VGV). This feature allows the AC signal component to swing around the middle of the 0-3.3V range (rail to rail, from minimum to maximum value), considering that a negative voltage at the output (with respect to ground reference) is not possible since operational amplifiers (OAMPS) have a unipolar power supply (0 to 3.3V). In addition, low-value output impedances are needed to allow voltage amplitude to be maintained and avoid current losses from the input signal when the electrochemical cell is connected. The above conditions are met by connecting the output of the high pass filter to an OAMP which acts as a buffer of the filtered signal to achieve a lower output impedance (less than 1 Ω) than the 1 KΩ of the AD5933 VOUT pin. In this way, using the above circuit, only one OAMP is needed to attain filtering, amplitude reduction, VGV, and low output impedance of the excitation signal. Finally, to isolate high-frequency harmonics from wireless communication and the AD5933 MCLK itself, a 100 KHz two-pole SKLPF was implemented in a second OAMP circuit. The chosen OAMP IC was the AD8608 IC, as it features four OAMPs circuits and allows precise low noise CMOS Rail-to-Rail amplification and filtering in low power applications such as the one from this work.

**PCB designs**


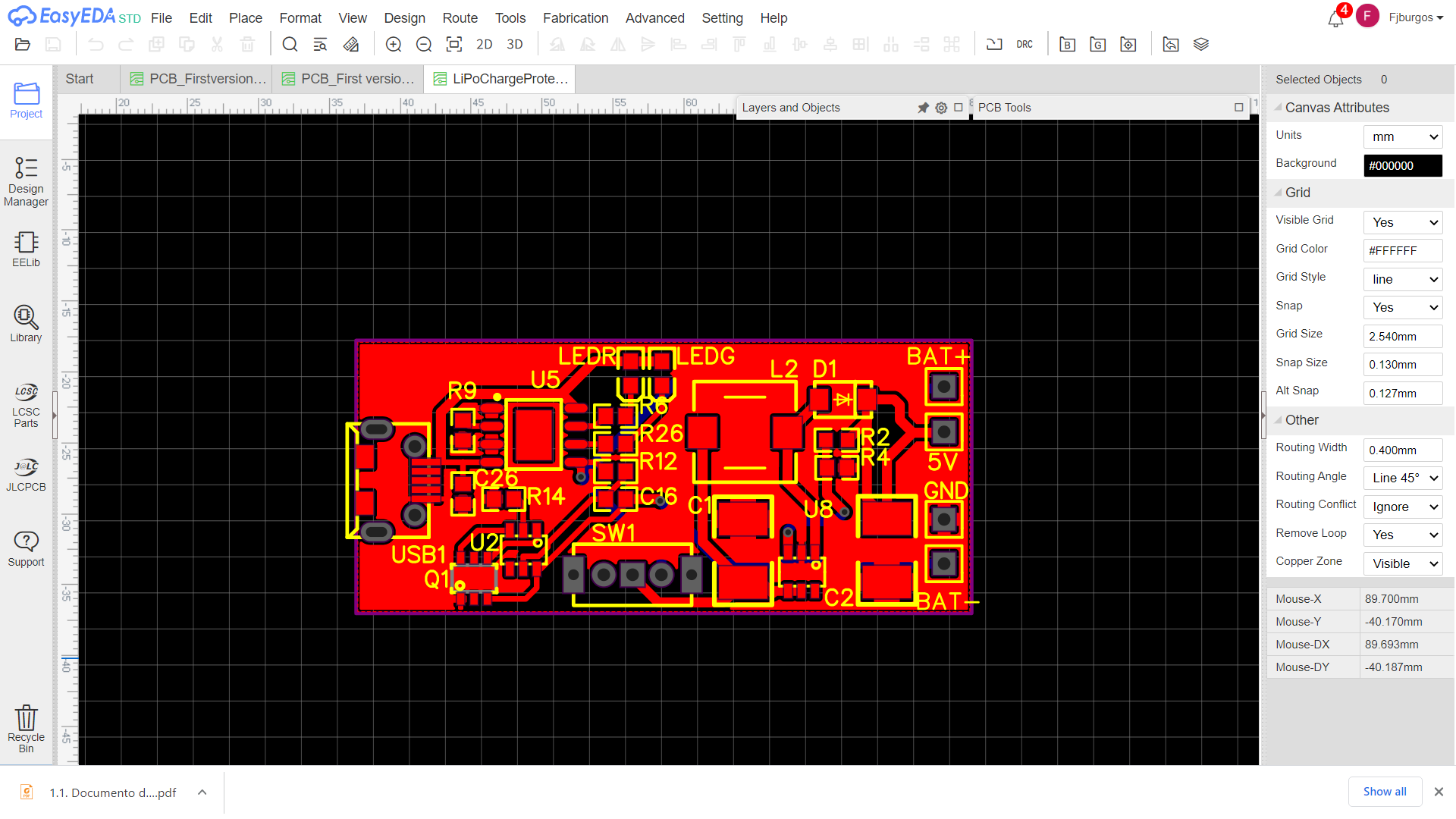


**Fig D**. PCB design of LiPo Battery boost/USB charger module. Top layer is shown in red, bottom layer in blue, top silk layer in yellow. Original design.


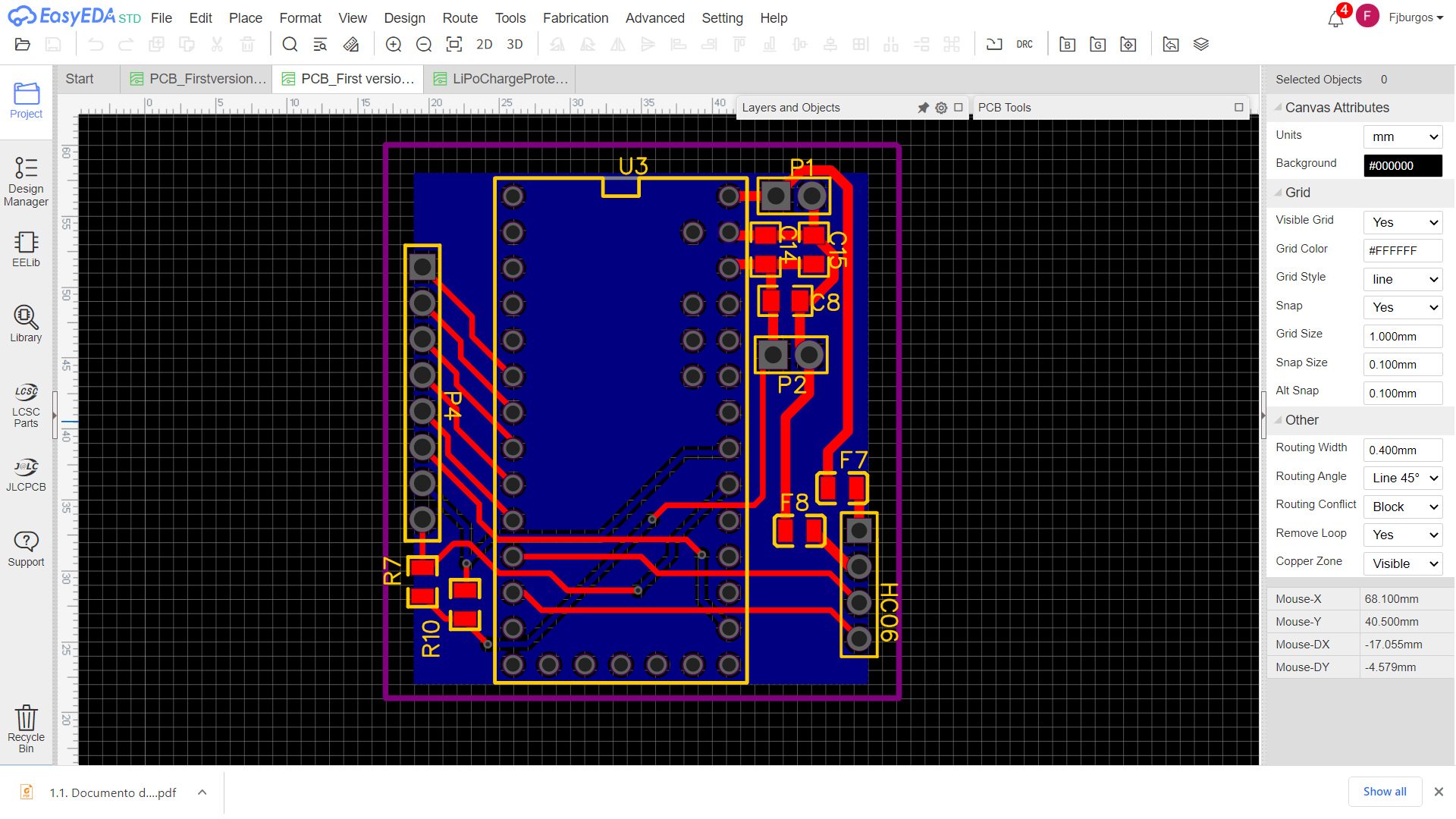


**Fig E**. PCB design of MCU module. Top layer is shown in red, bottom layer in blue, top silk layer in yellow. Original design.


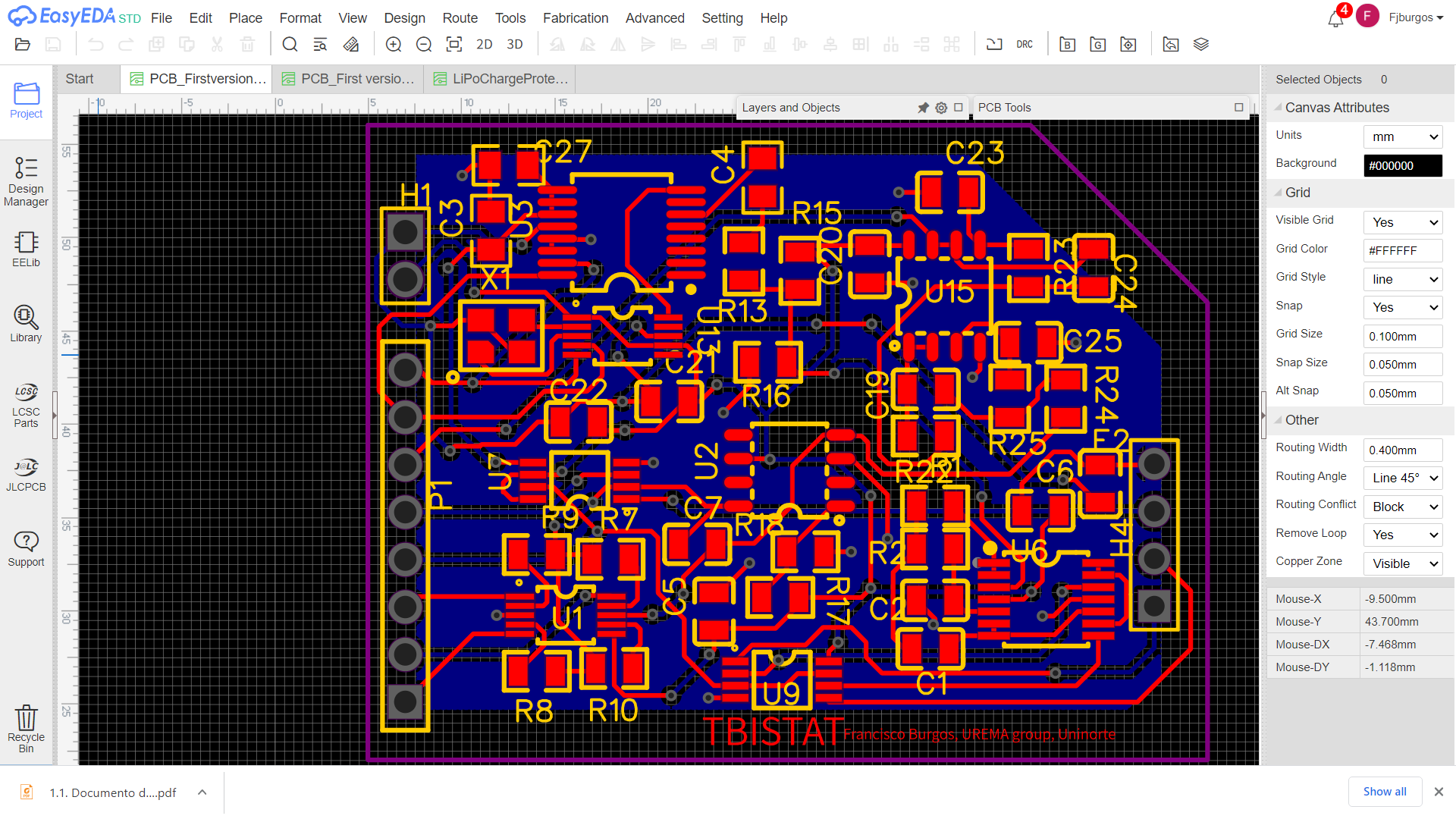


**Fig F**. PCB design of PC module. Top layer is shown in red, bottom layer in blue, top silk layer in yellow. Original design.

**References**

1. Zhang D, Lu Y, Zhang Q, Liu L, Li S, Yao Y, et al. Protein detecting with smartphone-controlled electrochemical impedance spectroscopy for point-of-care applications. Sensors Actuators, B Chem. 2016;222: 994–1002. doi:10.1016/j.snb.2015.09.041

2. Jenkins DM, Lee BE, Jun S, Reyes-De-Corcuera J, McLamore ES. ABE-STAT, a fully open-source and versatile wireless potentiostat project including electrochemical impedance spectroscopy. J Electrochem Soc. 2019;166: B3056–B3065. doi:10.1149/2.0061909jes

3. Chabowski K, Piasecki T, Dzierka A, Nitsch K. Simple Wide Frequency Range Impedance Meter Based on AD5933 Integrated Circuit. Metrol Meas Syst. 2015;22: 13–24. doi:10.1515/mms-2015-0006

4. Devices A. 1 MSPS, 12-Bit Impedance Converter, Network Analyzer. 2012.

5. Devices A. Evaluation Board User Guide UG-364. 2013.

6. Al-Ali A, Elwakil A, Ahmad A, Maundy B. Design of a portable low-cost impedance analyzer. BIODEVICES 2017 - 10th Int Conf Biomed Electron Devices, Proceedings; Part 10th Int Jt Conf Biomed Eng Syst Technol BIOSTEC 2017. 2017;2017-Janua: 104–109. doi:10.5220/0006121901040109

7. Devices A. Evaluation Board for the 1 MSPS 12-Bit Impedance Converter Network Analyzer. 2013.

8. Matsiev L. Improving performance and versatility of systems based on single-frequency dft detectors such as AD5933. Electron. 2014;4: 1–34. doi:10.3390/electronics4010001

9. Hernández H, Ruiz Reynoso A, Trinidad JC, González, Carlos O. Miranda JG, Mandujano AMR, Hernández J, et al. Electrochemical Impedance Spectroscopy (EIS): A Review Study of Basic Aspects of the Corrosion Mechanism Applied to Steels. intechopen. 2019. Available: http://dx.doi.org/10.5772/intechopen.94470

10. Victoria SN, Ramanathan S. Effect of potential drifts and ac amplitude on the electrochemical impedance spectra. Electrochim Acta. 2011;56: 2606–2615. doi:https://doi.org/10.1016/j.electacta.2010.12.007
