# Supplementary material for: TBISTAT: An open-source, wireless portable, electrochemical impedance spectroscopy capable potentiostat for the point-of-care detection of S100B in plasma samples
Source: PLoS One. 2022 Feb 7;17(2):e0263738. doi: 10.1371/journal.pone.0263738 (PMC8820642; doi:10.1371/journal.pone.0263738)
Supplement: S2 File — Additional details concerning software design. (DOCX) [file pone.0263738.s002.docx]

**S2. Software design**

Figure A shows the overall functionality of each TBISTAT component. An android application was developed to interface with the MCU firmware over either B2.0 or BLE protocols.


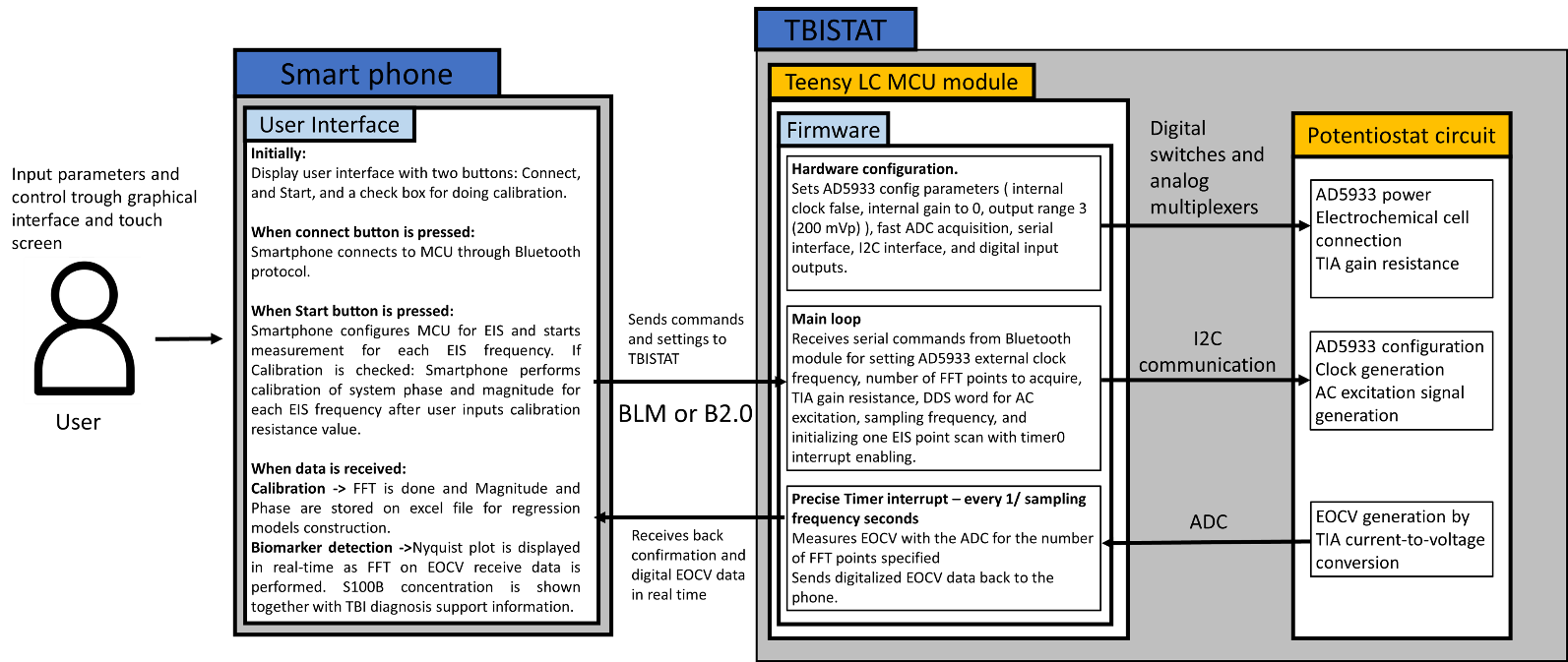


**Fig A**. Block diagram of TBISTAT components and functionality.

**MCU firmware**

**Initial configuration**

A teensy LC board was used as MCU for the TBISTAT. The firmware initially sets ADC, serial, I2C, Si551, AD5933, interrupt, and digital input/outputs configuration. A fast and accurate implementation of continuous ADC is achieved using Pedro Villanueva's implementation [1]. I2C communication is programmed using the Arduino wire library, which allows easy I2C configuration [2]. Similarly, the serial interface is programmed using serial library functions [3]. AD5933 is programmed using a modified version of Michael Meli AD5933 library [4]. Similarly, SI5351 configuration is done using the Etherkit SI5351 library [5]. Finally, fast, accurate time interrupts for ADC of EOC are achieved using the PJRC interval timer library [6].

**Main loop**

Using the firmware developed for RFDUINO by Ainla et al. [7] as a reference, the Teensy LC main loop is equipped to listen, interpret, and execute the commands sent by the smartphone. The teensy LC communicates with smartphones by sending and receiving commands in the string format, in the form x("number"), which are received and sent by the BM. The letter x is the command code that denotes a single uppercase character in the range [A-Z], and the string "number" represents a numerical value in long type when converted in the MCU firmware (32-bit signed integer). The MCU serial module receives serial commands in the above format from the android application for setting AD5933 external clock frequency, number of FFT points to acquire, TIA gain resistance, DDS word for AC excitation (see AD5933 library and [8–10]), sampling frequency, and initializing one EIS point scan with timer0 interrupt. Once TBISTAT receives the command and executes it, it sends the command "G" to acknowledge confirmation of the performed action. Table 1 shows the description of the input commands used in the MCU firmware. Once command "F" is received by the MCU, serial communication is stopped, and AC excitation by AD5933 begins.

Table 1. Input/output commands used for communication between MCU firmware and the smartphone application.

| Char | Parameter | Function | Description |
| --- | --- | --- | --- |
| A | 1 to 4 | TIA amplifier resistance | Selects between 10k, 100k, 1M, or 10M amplifier resistance for TIA |
| B | 1-500000 | AD5933 excitation frequency word | DDS word for choosing excitation frequency in AD5933 |
| C | 1-50000 | Timer interrupt period | Time in microseconds after which interrupt occurs |
| D | 1 to 5000 | Number of FFT measurement points | number of FFT sample points |
| E | 10000-16000000 | External clock frequency | SI5351 clock definition for AD5933 external clock |
| F | none | Start AC excitation in AD5933 | Begins AC excitation by AD5933. Enable Timer0 interrupt, disable serial communication. |
| G | none | Action is completed | Sends confirmation of completed command |
| H | 0-65535 | EOC ADC value for each FFT point | Sends one digitized EOC ADC data point value from the MCU to the Android application |
| M | None | Scan completed | ADC of all FFT points have been made, and data has been sent to the Android application. |

**Precise Timer interrupt/ADC**

The sampling time (sampling frequency) was appropriately defined to obtain a frequency resolution that produced FFT frequency bins whose integer multiplication resulted in a number as close as possible to the AC excitation frequency of each of the 36 EIS frequency points. Thus, spectral leakage to adjacent frequency bins was greatly diminished during the FFT of digitized EOC.

The timer0 interrupt was used in the Teensy LC firmware for achieving C(parameter) microseconds interruptions. It measures EOC with the ADC during its execution and stores 12-bits unsigned integers for each FFT point in a buffer (0 to 4095). After D(parameter) points have been acquired, the timer0 interrupt is stopped, serial communication is reestablished, and each sample point in the buffer is type casted to a long variable for long-to-string conversion in the format "HnumberF" and sent via the serial communication with the BM. This format allows easy string-to-double conversion in the android application for FFT calculations. Once all FFT points have been sent, the MCU sends the character "m," indicating that the scan for the defined frequency has finished.

**Android application**

**Bluetooth communication**

Serial Bluetooth Classic protocol and BLE were implemented using Douglas Roma's library [11].

**User interface**

The user interface comprises only two buttons (connect and start), a checkbox for calibration, and a text input space for selecting calibration resistance value. The user initially presses the connect button to establish a Bluetooth connection between the smartphone and the BM connected to the MCU. When the start button is pressed, the smartphone configures MCU for EIS and starts measuring each frequency point. If the Calibration checkbox is checked before pressing start, the user must input a number corresponding to the calibration resistance to be used. Then, the smartphone performs calibration of system phase and magnitude for each EIS frequency for the defined calibration resistance. When data coming from BM is received, an FFT is performed. If the user is performing calibration, the magnitude and phase are stored on an excel file. The creation, update, writing, and reading of excel files was implemented using Ranit Raj excel creation and editing libraries [12]. The calibration file is updated every time a new calibration measurement is done. Once this process is performed for all excitation frequencies of the EIS, the user is asked to change calibration resistance and perform another round of calibration for the new chosen resistance. Suppose the user is performing a conventional measurement in a two or three-electrode system to detect the S100B biomarker. In that case, a Nyquist plot is displayed in real-time as FFT on each EOC receive data is performed. Once EIS is finished, ΔRCT of the EIS and ΔC from SFA are calculated for finding S100B concentration, which is shown together with TBI diagnosis support information.

**FFT**

A 1000-point FFT on incoming digitized EOC data was performed using Piotr Wendykier FTT implementation [13]. A total of 36 logarithmically spaced frequency points were chosen between the 1-10 KHz frequency range. Sampling times and AD5933 MCLK values were carefully selected to obtain a frequency resolution that reduced spectral leakage in the FFT by producing frequency bins which were almost exact integer multiplications of the measured signal frequency. The FFT algorithm produces an n-size vector, where n is the number of points acquired. The function outputs half of the real DFT, considering the symmetry condition. The real and imaginary parts for each frequency bin were found using:

| $Re\left[ k \right]=FFT\left[ 2*k \right] , 0\leq k<\frac{n}{2}$ | (1) |
| --- | --- |

| $Im\left[ k \right]=FFT\left[ 2*k+1 \right],0<k<\frac{n}{2}$ | (2) |
| --- | --- |

Where k is an index that moves along the frequency bins. The frequency bin corresponding to the acquired AC signal corresponds to the highest FFT magnitude. Each EIS scan is performed five times, and a median filter using the bubble sort algorithm is employed to diminish errors coming from signal distortion and the intrinsic system variability. The chosen FFT magnitude and phase values are stored on position three of the output bubble sort vector, and are employed for finding the impedance magnitude and phase of the measured impedance.

**Nyquist plot construction**

Real-time display of impedance measurements in the Android application is performed using the Android chart implementation made by Philipp Jahoda [14]. Each point of the Nyquist plot is displayed on the quadrant one of a coordinate plane with real impedance component as abscissa and impedance imaginary component as ordinate. The real and imaginary components of the impedance measurement for each frequency point are given by:

| $real value=IM*cos(phase)$ | (3) |
| --- | --- |

| $Imaginary value=IM*sin(phase)$ | (4) |
| --- | --- |

Where IM corresponds to the impedance magnitude.

**TBISTAT calibration**

After EIS is performed, a 648 (6 resistors per TIA resistance x 3 TIA resistances x 36 frequency points) x 5 matrix is populated with calibration resistor known impedance magnitude (between 103.5 Ω and 33200 Ω), frequency value (between 1 Hz and 10 KHz) point of AC excitation signal, TIA resistor (10 KHz, 100 KHz or 1MHz), FFT magnitude, and FFT phase for each defined setting. This matrix is stored in an excel file created by the android application, which can be read or modified in the event of another calibration or EIS measurement of an unknown S100B concentration in the defined biosensor platform. FFT magnitude and phase are given by:

| $FFT magnitude=\sqrt{Re\left[ k \right]^{2}+Im\left[ k \right]^{2}}$ | (5) |
| --- | --- |

| $FFT phase=\mathrm{atan} \frac{Im\left[ k \right]}{Re\left[ k \right]}$ | (6) |
| --- | --- |

**Impedance measurement**

The default program setting of the TBISTAT is to perform a complete EIS of the unknown impedance, unless calibration checkbox is selected by the user. The TBISTAT selects the lowest TIA amplifier resistance and the highest AC excitation frequency, corresponding to 10 KΩ and 10 KHz, respectively. After excitation, ADC takes place, digitized EOCV data is transferred to the smartphone application, FFT is performed, and FFT magnitude and phase are calculated for the desired frequency point. The following code is executed for the obtention of impedance magnitude and phase:

if (FFT magnitude> high FFT magnitude limit)

"Impedance is too small to be measured."

else if (FFT magnitude>low FFT magnitude limit)

- Interpolate ${IM}_{i}$ and ${IP}_{i}$
- Update Nyquist plot

else if (FFT magnitude<low FFT magnitude limit AND TIA amplifier resistor!=10MΩ)

- Change TIA amplifier resistance to next value
- Repeat frequency point impedance measurement

else

"Impedance is too high to be measured."

- Finish Nyquist plot.

Where IM and IP correspond to the impedance magnitude and phase, respectively. Low and high FFT magnitude limits for each frequency point correspond to the lowest and highest FFT magnitude values for each TIA resistance setting previously found and stored during calibration.

The impedance magnitude is found by interpolation using the magnitude and phase values of the current FFT and the magnitude and phase values of the calibration resistances in the current setting, i.e., 10 KHz TIA resistance, 10KHz frequency, 103.5 Ω to 1183 Ω resistor range. The impedance phase is the absolute value of the difference between the system phase defined using the same method as above (pairing FFT magnitude with system phase as the unknown value in the interpolation) and the measured FFT phase for the scanned EIS frequency point.

**References**

1. Villanueva P. Teensy ADC. 2020. Available: https://github.com/pedvide/ADC

2. Arduino. Wire library. 2010. Available: https://www.arduino.cc/en/reference/wire

3. Arduino. Serial Library. 2010. Available: https://www.arduino.cc/reference/en/language/functions/communication/serial/

4. Melli M. AD5933 Arduino Library. 2017. Available: https://github.com/mjmeli/arduino-ad5933

5. Etherkit. Si5351 Library for Arduino. 2019. Available: https://github.com/etherkit/Si5351Arduino

6. PJRC. Teensy Interval timer. 2019. Available: https://www.pjrc.com/teensy/td_timing_IntervalTimer.html

7. Ainla A, Mousavi MPS, Tsaloglou M-N, Redston J, Bell JG, Fernández-Abedul MT, et al. Open-Source Potentiostat for Wireless Electrochemical Detection with Smartphones. Anal Chem. 2018;90: 6240–6246. doi:10.1021/acs.analchem.8b00850

8. Devices A. 1 MSPS, 12-Bit Impedance Converter, Network Analyzer. 2012.

9. Devices A. Evaluation Board User Guide UG-364. 2013.

10. Devices A. Evaluation Board for the 1 MSPS 12-Bit Impedance Converter Network Analyzer. 2013.

11. Roma D. AndroidBluetoothLibrary. 2018. Available: https://github.com/douglasjunior/AndroidBluetoothLibrary

12. Raj R. Creating an excel in android. 2020. Available: https://github.com/ranitraj/ContactsEntrepot

13. Wendykier P. JTransforms. 2014. Available: https://github.com/wendykierp/JTransforms

14. Jahoda P. MPAndroidChart. 2019. Available: https://github.com/PhilJay/MPAndroidChart
