# Supplementary material for: TBISTAT: An open-source, wireless portable, electrochemical impedance spectroscopy capable potentiostat for the point-of-care detection of S100B in plasma samples
Source: PLoS One. 2022 Feb 7;17(2):e0263738. doi: 10.1371/journal.pone.0263738 (PMC8820642; doi:10.1371/journal.pone.0263738)
Supplement: S7 File — (DOCX) [file pone.0263738.s007.docx]

**S7. Statistical test and figures**

**
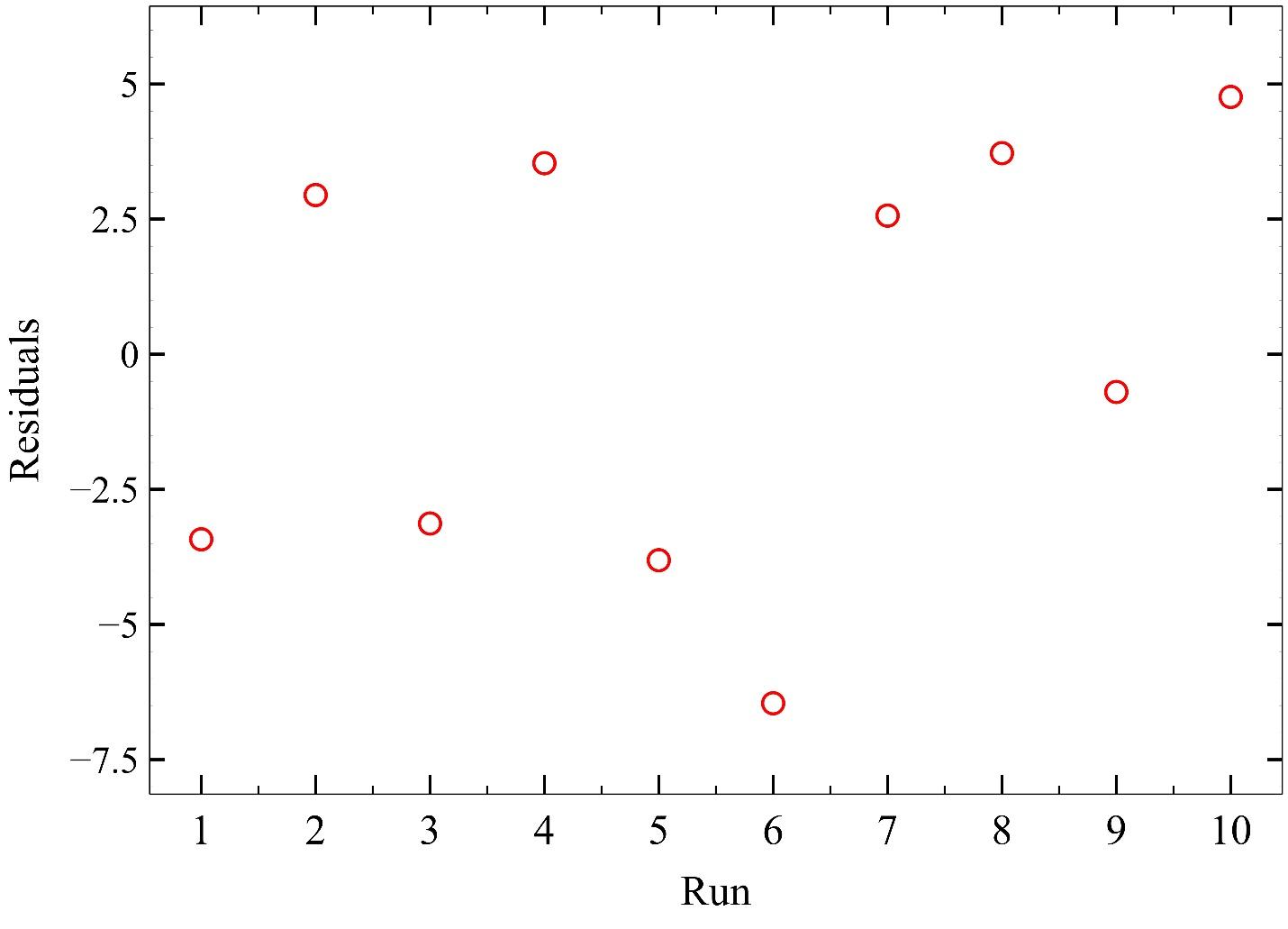
**

**Fig A**. Residuals plot for T test. No structure is found on the residuals; hence, they can be thought of as independent.

Table 1. Delta RCT (ΔRCT) for each experimental run.

| **Run** | **Log[S100B]** | **ΔRCT change (𝛀)** |
| --- | --- | --- |
| 7 | 1.5 | 2984 |
| 1 | 1.5 | 2543 |
| 4 | 1.5 | 3126 |
| 8 | 1.5 | 3690 |
| 9 | 2 | 5903 |
| 3 | 2 | 8578 |
| 5 | 2 | 7448 |
| 12 | 2 | 9351 |
| 2 | 2.5 | 17257 |
| 11 | 2.5 | 20364 |
| 10 | 2.5 | 20380 |
| 6 | 2.5 | 18074 |


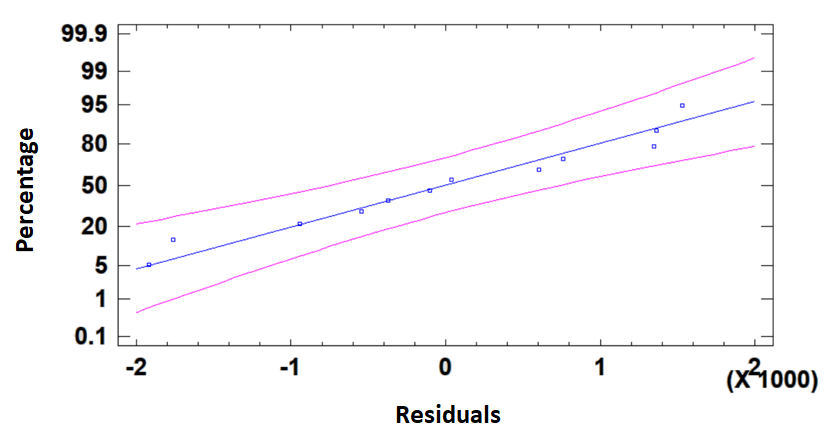


**Fig B**. Graphical verification of the assumption of normality for the S100B detection experiment.


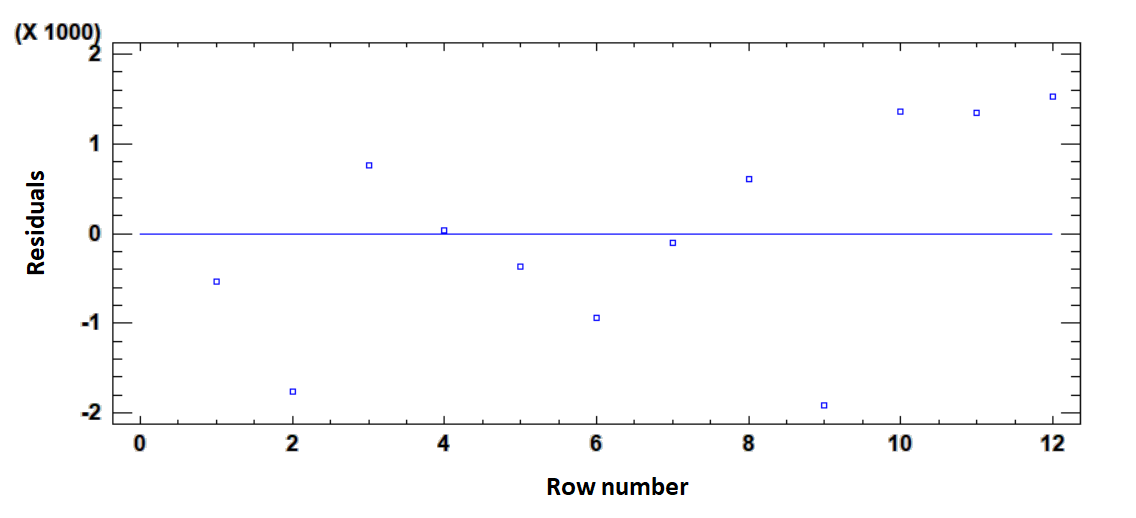


**Fig C**. Graphical verification of the assumption of independence for the S100B detection experiment.

**Table 2**. Statistical quantitative tests and analysis of variance for the S100B detection experiment. Homoscedasticity is not fulfilled. Therefore, a Welch test is performed for non-homogeneity of variance between groups. Games-Howell test shows statistically significant differences between groups.

| **Test** | **Statistic** | **P-Value** |
| --- | --- | --- |
| Shapiro-Wilk | 0.940825 | **0.508837** |
| Levene's | 5.13619 | **0.0325** |

| **Test** | **Statistic** | **Gl1** | **Gl2** | **P-Value** |
| --- | --- | --- | --- | --- |
| Welch test | 168.393 | 2.00 | 4.65 | **0.0000** |

| **Games-Howell test** | | | |
| --- | --- | --- | --- |
| **Log (S100B)** | **Cases** | **Mean** | **Homogeneous groups** |
| 1.5 | 4 | 3085.75 | X |
| 2 | 4 | 7820.0 | X |
| 2.5 | 4 | 19018.8 | X |

| **Contrast** | **Sig.** | **Difference** | **+/- Limits** |
| --- | --- | --- | --- |
| 1.5 - 2 | * | **-4734.25** | 2951.11 |
| 1.5 - 2.5 | * | **-15933.0** | 3163.63 |
| 2 - 2.5 | * | **-11198.8** | 3364.12 |

| **Log[S100B]** | **Mean (Ω)** | **SD** | **RSD (%)** |
| --- | --- | --- | --- |
| 1.5 | 3085.877 | 473.2383 | 15.33562 |
| 2 | 7819.996 | 1498.22 | 19.15883 |
| 2.5 | 19018.61 | 1597.916 | 8.401854 |

**Table 3**. Assessment of the reproducibility of the response variable (∆RCT) in AUIDEs through the estimation of the relative standard deviation (RSD).

**Table 4**. Assessment of the regression model suitability for the response variable (∆RCT) vs. S100B concentration.

| **Coefficients** | | | | |
| --- | --- | --- | --- | --- |
| **Parameter** | **Estimated Minimum Square** | **Standard error** | **T Statistic** | **P-Value** |
| Intercept | 1789.73 | 594.922 | 3.00835 | **0.0132** |
| Slope | 54.9336 | 3.0954 | 17.7468 | **0.0000** |

| **Analysis of Variance** | | | | | |
| --- | --- | --- | --- | --- | --- |
| **Sov** | **Sum of Squares** | **DoF** | **Mean Square** | **F-ratio** | **P-value** |
| Model | 5.33698E8 | 1 | 5.33698E8 | 314.95 | **0.0000** |
| Residual | 1.69455E7 | 10 | 1.69455E6 |  |  |
| Total (Corr.) | 5.50643E8 | 11 |  |  |  |


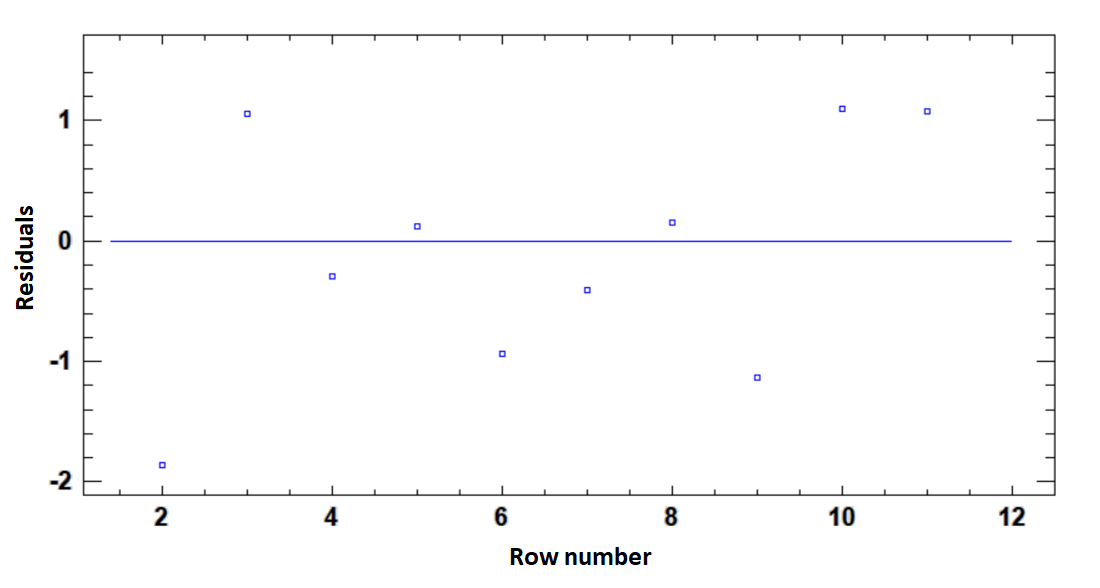


**Fig D**. Graphical verification of the assumption of independence for the S100B regression model.

**Table 5**. Lack-of-fit test of the regression model for the response variable (∆RCT) vs. S100B concentration.

| **SoV** | **Sum of Squares** | **DoF** | **Mean Square** | **F-ratio** |
| --- | --- | --- | --- | --- |
| Model | 5.33698E8 | 1 | 5.33698E8 | 314.95 |
| Residual | 1.69455E7 | 10 | 1.69455E6 |  |
| Lack of fit | 1.88303E6 | 1 | 1.88303E6 | 1.13 |
| Pure error | 1.50625E7 | 9 | 1.67361E6 |  |
| Total (Corr.) | 5.50643E8 | 11 |  |  |

| **SoV** | **P-Value** |
| --- | --- |
| Model | **0.0000** |
| Residual |  |
| Lack of fit | **0.3165** |
| Pure error |  |
| Total (Corr.) |  |
